# Src Kinases Regulate *De Novo* Actin Polymerization during Exocytosis in Neuroendocrine Chromaffin Cells

**DOI:** 10.1371/journal.pone.0099001

**Published:** 2014-06-05

**Authors:** María José Olivares, Arlek M. González-Jamett, María José Guerra, Ximena Baez-Matus, Valentina Haro-Acuña, Narcisa Martínez-Quiles, Ana M. Cárdenas

**Affiliations:** 1 Centro Interdisciplinario de Neurociencia de Valparaíso, Facultad de Ciencias, Universidad de Valparaíso, Playa Ancha, Valparaíso, Chile; 2 Departamento de Microbiología (Inmunología), Facultad de Medicina, Universidad Complutense de Madrid, Madrid, Spain; Hungarian Academy of Sciences, Hungary

## Abstract

The cortical actin network is dynamically rearranged during secretory processes. Nevertheless, it is unclear how *de novo* actin polymerization and the disruption of the preexisting actin network control transmitter release. Here we show that in bovine adrenal chromaffin cells, both formation of new actin filaments and disruption of the preexisting cortical actin network are induced by Ca^2+^ concentrations that trigger exocytosis. These two processes appear to regulate different stages of exocytosis; whereas the inhibition of actin polymerization with the N-WASP inhibitor wiskostatin restricts fusion pore expansion, thus limiting the release of transmitters, the disruption of the cortical actin network with cytochalasin D increases the amount of transmitter released per event. Further, the Src kinase inhibitor PP2, and cSrc SH2 and SH3 domains also suppress Ca^2+^-dependent actin polymerization, and slow down fusion pore expansion without disturbing the cortical F-actin organization. Finally, the isolated SH3 domain of c-Src prevents both the disruption of the actin network and the increase in the quantal release induced by cytochalasin D. These findings support a model where a rise in the cytosolic Ca^2+^ triggers actin polymerization through a mechanism that involves Src kinases. The newly formed actin filaments would speed up the expansion of the initial fusion pore, whereas the preexisting actin network might control a different step of the exocytosis process.

## Introduction

The subplasmalemmal actin mesh plays a pivotal function during the secretory process in neuroendocrine cells. The first studies conducted to understand the role of cortical actin filaments (F-actin) in exocytosis proposed that it constitutes a physical barrier that restricts the secretory vesicle access to the plasma membrane. Consistent with this idea, an uninterrupted cortical actin ring is observed in non-stimulated cells, while this actin ring is disrupted upon stimuli that increase cytosolic Ca^2+^ concentrations and trigger exocytosis [1]. Later findings showed that F-actin is rearranged upon cell stimulation to favor the release of transmitters [2]. Indeed, the actin nucleation promoting factor (NPF) neural Wiskott-Aldrich syndrome protein (N-WASP) is recruited upon stimulation in PC12 cells, where it seems to mediate the secretory response [3]. It was also observed that F-actin forms trails that favor the secretory vesicle motion to the plasma membrane [4] and that the subplasmalemmal actin network regulates the expansion of the fusion pore [5]–[6], an intermediate structure formed during the exocytotic process [7], and the amount of catecholamine released per individual events [6], [8]. Nevertheless, in these latter reports [5]–[6], [8], the role of F-actin was evaluated using pharmacological agents that indiscriminately disrupt the new F-actin formation and the preexisting cortical actin network, making it difficult to separate the differential roles of *de novo* actin polymerization from the preexistent actin mesh during exocytosis.

Actin polymerization and depolymerization require the concerted action of actin regulatory proteins whose activity needs to be finely tuned by protein kinases. Among them are Src kinases, a family of non-receptor tyrosine kinases that phosphorylate and regulate the activity of NPFs such as cortactin [9]–[10] and N-WASP [11] and severing-factors such as cofilin [12] and gelsolin [13]–[14].

Src- kinase family comprises nine proteins: c-Src, c-Yes, Fyn, c-FGR, Lck, Hck, Blk, Lyn and Yrk, which share a common structure that includes: a SH1 domain with tyrosine kinase activity, a SH2 domain that binds phosphotyrosines, a SH3 domain that targets proline-rich regions in different partners, and a SH4 domain containing myristylation signals that guide Src to membranes [15]–[16]. The SH3 domain seems to be the region responsible for the Ca^2+^-mediated regulation of some Src kinase members [17]. In this regard, a rise in the cytosolic Ca^2+^ concentration inhibits c-Yes but promotes c-Src [17]–[18] and Fyn [19] activation. Furthermore, Ca^2+^-induced activation of c-Src reportedly mediates Ca^2+^-dependent processes such as neurite growth [20] and neurotransmitter release [21]–[22], suggesting that some Src kinase members may constitute key regulatory molecules of actin polymerization during exocytosis.

In the present work, we address the hypothesis that Src kinases regulate the exocytotic release of transmitters by controlling the cortical actin dynamics by using neuroendocrine adrenal chromaffin cells (ACCs). These cells constitute a widely used model to study the exocytotic release of neurotransmitters and hormones. Moreover, they reportedly express three Src family members: c-Src, c-Yes and Fyn [19], with c-Src found in both plasma membrane and secretory vesicles, and Fyn localized mainly in the plasma membrane [19], [23].

The data presented here show that both formation of new actin filament and disruption of preexisting cortical actin are induced by Ca^2+^ concentrations that trigger exocytosis in ACCs. These two processes appear to regulate different stages of the exocytosis; while the newly formed F-actin is involved in the expansion of the fusion pore, the preexisting actin seems to control a later step. In this mechanism, Src kinases appear as key regulators of the Ca^2+^-dependent actin polymerization and the fusion pore expansion.

## Materials and Methods

### Chromaffin cells culture

Bovine ACCs were isolated and cultured as previously described [24]. Briefly, bovine suprarenal glands obtained from a local slaughterhouse, were perfused with a LOCKE/glucose solution (mM: 154 NaCl, 5.6 KCl, 3.6 NaHCO3, 10 Hepes, 100 U/mL penicillin, 100 µg/ml gentamycin at pH 7.4) and then digested in the presence of 0.25% collagenase (Roche)/0.01% trypsin inhibitor (Gibco BRL) and 0.5% bovine serum albumin (BSA). After digestion, the adrenal medulla was crumbled and filtered through a surgical gauze filter. The resulting cell suspension was subjected to a percoll gradient and the ACC enriched fraction was resuspended in a 1∶1 mixture of Dulbecco's modified F-12 medium (Gibco BRL) supplemented with 10% of fetal bovine serum (Gibco), and cultured at a density of 3×10^5^ cells/ml in 0.01% poly-L-lysine (Sigma) treated dishes. Cultured ACCs were kept at 37°C, 5% CO_2_ and 95% air for at least 24 hours prior to experiments.

### Cloning, expression and purification of c-Src-SH2 and c-Src-SH3 GST-fusion proteins

The pcDNA3 vector containing the chicken Src cDNA was kindly provided by Dr. Ligia Toro (California University, Los Angeles, USA). The vector was linearized using HindIII and Not I enzymes and the region encoding SH2 and SH3 domains of c-Src were amplified by PCR using specific primers (forward: 5′ CTT GGA TCC CAG GCT GAA GAG TGG TAC 3′ and reverse: 5′ GAT GAA TTC GCA GAC GTT GGT CAG 3′ for SH2 domain); (forward: 5′ CTT GGA TCC GGC GGC GTC ACC ACT TTC GTG 3′ and reverse: 5′ GCG GAA TTC GAT GGA GTC TGA GGG CGC GAC ATA 3′ for SH3 domain). The resulting PCR products were digested with BamHI and EcoRI, subcloned into the glutathione-S-transferase (GST) fusion vector pGEX4T-2, expressed in *Escherichia coli* BL21 cells and purified with glutathione agarose beads (Life Technologies) using standard protocols.

The GST fusion protein of the N-WASP proline-rich domain (PRD) contained into the pGEX-6P1 vector was previously described [25].

### Intracellular injections and pharmacological treatments

To evaluate the role of Src kinases, cells were incubated during 20 minutes at 37°C with 10 µM of the Src kinase inhibitor PP2 or its inactive analogue PP3 (Sigma Aldrich). To acutely inhibit SH2 or SH3 domain-dependent Src kinase association to different partners, cultured ACCs were injected with 5 µM of SH2 or SH3-GST fusion proteins (or GST alone), using an InjectMan system (Eppendorf) and 0.5 µm- diameter femtotips (Eppendorf) and 30 minutes later monitored by amperometry. To suppress N-WASP activation, cultured ACCs were incubated with 5 µM of wiskostatin (Calbiochem) for 10 minutes. To disrupt actin network, cells were incubated with 4 µM of cytochalasin D (Gibco) during 10 min at 37°C.

### F-actin formation and actin cytoskeleton organization in chromaffin cells

The new formation of actin fibers was assessed in cultured ACCs permeabilized during 6 minutes with 20 µM digitonin in KGEP buffer (mM: 139 K-glutamate, 20 PIPES, 5 EGTA, 2 ATP-Mg^2+^, pH 6.6), in the presence of 0.3 µM Alexa Fluor 488 (or Alexa Fluor 554) G-actin conjugate (Life Technologies) and different free Ca^2+^ concentrations. After permeabilization cells were fixed for 15 minutes at 4°C with 4% paraformaldehyde (PFA) and visualized in a confocal microscope (Eclipse Nikon80i; 3 excitation lasers: 408 nm, 488 nm and 543 nm).

To evaluate the effects of the different reagents on the preexisting cortical actin network, cultured ACCs were incubated in a Krebs-Hepes solution (mM: 140 NaCl, 5.9 KCl, 1.2 MgCl_2_, 2 CaCl_2_, 10 Hepes/Na, pH 7.4) during 20 minutes at 37°C and then fixed for 15 minutes at 4°C with 4% PFA. Cells were then washed with phosphate buffered saline (PBS) (mM: 137 NaCl, 2.7 KCl, 10 Na_2_HPO_4_, 1.8 KH_2_PO_4_, pH 7.4), permeabilized with 0.2% Triton-X-100 during 12 minutes at room temperature, then incubated during 40 minutes with 1 µM phalloidin-rhodamine B (Sigma Aldrich) and visualized by confocal microscopy (Eclipse Nikon 80i).

Confocal images were analyzed and processed using the Image-J software (NIH, USA). The cortical actin ring fluorescence (for *de novo* formed and pre-existing actin) was measured subtracting the mean fluorescence 1 µm under the cell periphery to the total cell fluorescence intensity.

### Amperometric detection of exocytosis

Amperometric recordings were performed as previously described [26] using 5 µm-diameter carbon fibers (Thornel P-55; Amoco Corp) and a patch clamp amplifier (EPC-10 USB; HEKA Electronics). The amperometric signal was low-pass filtered at 1 KHz and digitalized at 5 Hz with the acquisition software PatchMaster (HEKA Electronics). During the recording, cells were perfused with a Krebs-Hepes solution. Exocytosis was evoked by a 10 s pressure ejection of 20 µM of the Ca^2+^-ionophore ionomycin.

### Spike analysis and statistics

Amperometric spikes were analyzed using the software IGOR 4 (Wavemetrics) and macros specifically designed to filter, identify and analyze individual amperometric spikes. All used macros are available at: http://webpages.ull.es/users/rborges/. Only spikes with amplitudes >10 pA were used for analysis. The criteria to analyze foot signals was restricted to foot amplitudes >3 pA and foot durations >3 ms. Each amperometric parameter was statistically analyzed by taking the median values of the events from individual cells and then averaging these values per treatment group. For amperometric data and image analysis “n” refers to the number of tested cells. Statistical comparisons were performed utilizing the Kruskal-Wallis test for nonparametric data or ANOVA test for parametric data. Results are expressed as mean ± standard error of the mean (SEM).

### Pull-down assays

Plated ACCs were lysed at 4°C in lysis buffer (mM:150 NaCl, 20 Hepes, 5 EDTA, pH 7.4 and 1% Triton X-100 plus protease inhibitor cocktail) and fresh extracts were used for immunoprecipitation and pull-down experiments. For the pull-down assay, 100 µl of glutathione agarose beads (Life Technologies) were incubated with 100 µg of c-Src-SH2-GST, c-Src-SH3-GST, N-WASP-PRD-GST or GST alone for 4 hours at 4°C, blocked by 30 minutes with 10% BSA and then incubated with 800 µg of ACC extract, overnight at 4°C. Beads were washed three times in lysis buffer and bound proteins eluted with SDS sample buffer for immunoblotting analyses using specific antibodies against cortactin (Abcam), c-Src (Cell Signaling) and GST (Upstate).

### Ethics statement

The present work includes the use of samples from animals (bovine adrenal glands) obtained from a local slaughterhouse, Frigorific Don Pedro, certified by the Agriculture and Livestock Service of the Chilean Government (certificate number: 04.2.03.0002) and regularly inspected by a veterinarian of the Chilean Health Service. Transport, processing and elimination of the samples were carried out in strict accordance with the Article 86 of the Sanitary Regulations of the Chilean Government (Supreme decree N° 977/96).

The protocols described in this article were approved by a Committee of Bioethics and Biosafety of the Faculty of Science, University of Valparaíso, directed by Professor Juan Carlos Espinoza, on March 7, 2011.

## Results

### Ca^2+^-dependent actin polymerization and depolymerization in adrenal chromaffin cells

In order to evaluate the contribution of Src kinases to the Ca^2+^-dependent actin cytoskeleton dynamics in ACCs we first analyzed how different Ca^2+^ concentrations affect both F-actin polymerization and depolymerization. To accurately adjust the intracellular Ca^2+^ concentrations, these experiments were performed in permeabilized ACCs. We used three different Ca^2+^ concentrations: 0,1 µM that corresponds to the Ca^2+^ concentration at resting conditions [27], and 1 and 10 µM free Ca^2+^, which reportedly induce exocytosis in permeabilized ACCs [28].

**Figure 1:**
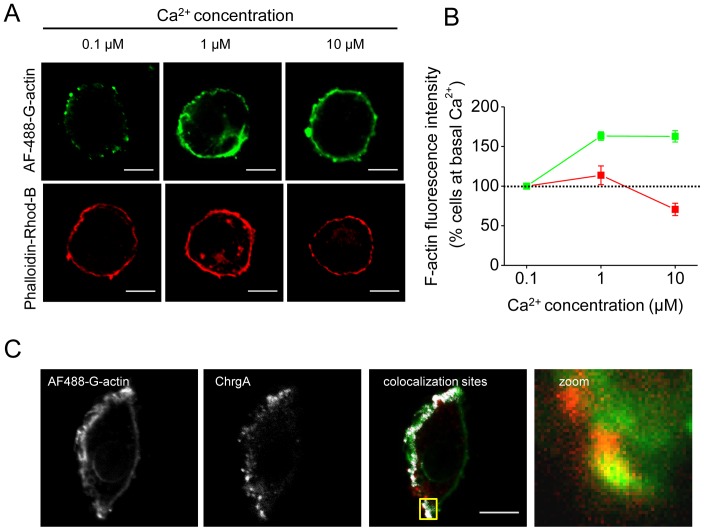
Ca^2+^-dependent cortical actin polymerization colocalizes with secretory granules in permeabilized chromaffin cells. (**A–B**) Cultured ACCs were permeabilized in KGEP buffer for 6 minutes with 20 µM digitonin in the presence of increasing concentrations of free Ca^2+^ and 0.3 µM of Alexa Fluor 488 G-actin, fixed and visualized by confocal microscopy (top panel). Under these conditions, the actin ring corresponds to recently polymerized actin filaments. To evaluate the effect of growing free Ca^2+^ concentrations on the preexisting cortical actin ring, digitonin-permeabilized ACCs were fixed and stained with 1 µM phalloidin-rhodamine B (bottom panel). (**B**) Quantification of the cortical actin fluorescence intensity shows the coexistence of assembly (green line) and disassembly (red line) of the cortical actin network at 10 µM of free Ca^2^ compared to basal Ca^2+^ levels (0.1 µM). Data are means ± SEM from 20–22 cells. (**C**) ACCs permeabilized in the presence of Alexa Fluor 488 G-actin and 10 µM of free Ca^2+^ were fixed and immunolabeled with a specific antibody against the chromaffin granule marker chromogranin A. F-actin and chromogranin A images were thresholded and colocalization sites were automatically detected (white spots). A colocalization site is highlighted in yellow. Note that the newly polymerized actin (green) colocalizes with chromogranin A (red). The mean score for the Pearson Correlation Coefficient between chromogranin A and F-actin at these sites was 0.89±0.03 (n = 12). Scale bar  = 10 µm.

As shown in Figure 1A a disruption of the subplasmalemmal actin ring stained with phalloidin-rhodamine B was observed at 10 µM of free Ca^2+^ (see bottom panel). However, a new formation of cortical actin filaments was also observed at 1 and 10 µM of free Ca^2+^ (see top panel), suggesting that actin polymerization coexists with a disassembly of the preexisting cortical actin network at Ca^2+^ concentrations that induce exocytosis in ACCs. Quantification of the cortical actin ring fluorescence intensity (Figure 1B) showed that at 10 µM Ca^2+^ the *de novo* cortical actin assembly increased by 50% with respect to that observed at 0.1 µM (green line), while the integrity of the cortical actin ring diminished with the higher Ca^2+^ concentration (red line).

To evaluate whether the newly formed F-actin associates with secretory vesicles, we performed a colocalization analysis between *de novo* polymerized F-actin and chromaffin granules immunolabeled with a specific antibody against the intragranular protein chromogranin A. As observed in Figure 1C, chromaffin granules colocalized in some specific sites with newly formed actin filaments. The mean score for the Pearson Coefficient between chromogranin A and F-actin at these sites was 0.89±0.03 (n = 12), an indicative value of a statistically significant correlation. These data suggest a role of the new actin polymerization during the exocytosis of secretory granules in ACCs.

### Src kinases control the de novo cortical actin assembly

Given that Src kinases are involved in both actin polymerization [9]–[10] and Ca^2+^-dependent transmitter release [21]–[22], our next step was to analyze the role of Src kinases on actin dynamics in both permeabilized and intact AACs. Permeabilized cells allow us to adjust the Ca^2+^ concentration, introduce fluorescent-tagged-G-actin, and separately evaluate the *de novo* F-actin formation from the F-actin network organization. In this condition, we did not observe a loss of Src kinases as compared with cortactin, a protein of a higher molecular weight (Figure S1), but we can not discard the leakage of smaller molecules involved in actin polymerization/depolymerization dynamics. On the other hand, intact cells allow us to visualize the general organization of the cortical F-actin network under resting or stimulated conditions.

**Figure 2:**
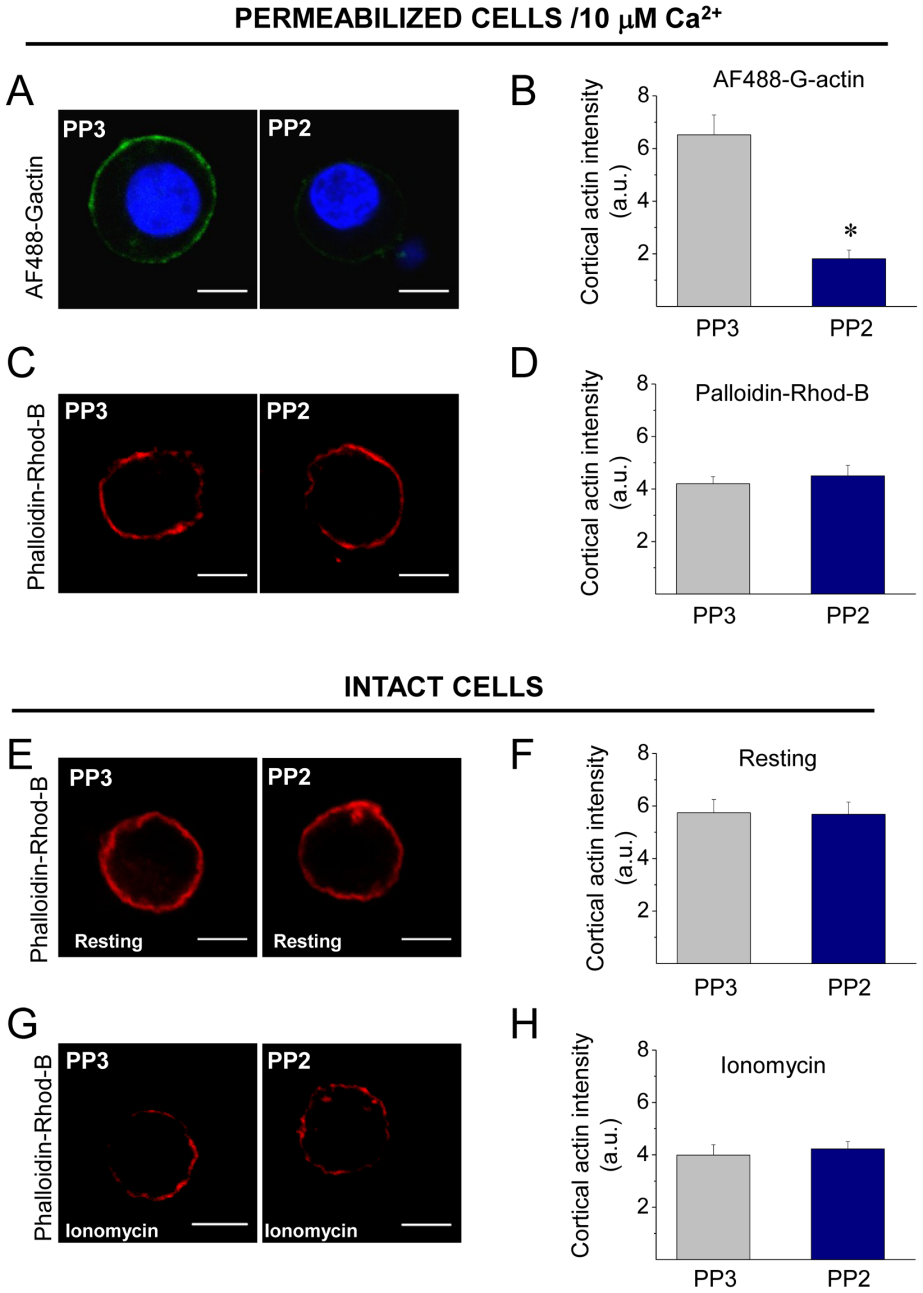
Inhibition of Src activity reduces Ca^2+^-dependent actin polymerization. (**A–B**) To analyze the impact of Src kinase inhibition on actin dynamics, ACCs were permeabilized in the presence of Alexa Fluor 488 G actin, 10 µM free Ca^2+^ and 10 µM of the Src inhibitor PP2 or its inactive analogue PP3. (**C–D**) To evaluate the drug effects on the organization of the cortical actin network, cells were permeabilized with digitonin for 6 minutes in the presence of PP2 (or PP3), then fixed and stained with 1 µM phalloidin-rhodamine B. (**E–H**) Intact cells were incubated for 20 min with 10 µM PP2 (or PP3), maintained in resting condition (E–F) or stimulated with 20 µM ionomycin for 10 s (G–H), then fixed and stained with 1 µM phalloidin-rhodamine B to confocal visualization. A, C, E and G show representative confocal images for each condition. Scale bar  = 10 µm. B, D, F and H correspond to the quantification of the cortical actin fluorescence intensity, where data are means ± SEM from at least 16 cells per each condition (*p<0.05 compared with PP3). Note that Src kinases inhibition with PP2 reduces the new formation of cortical actin filaments, but it does not affect the cortical actin network in permeabilized or intact cells.

As observed in Figure 2A, 10 µM of PP2 significantly reduced the new F-actin formation in cells permeabilized in the presence of 10 µM free Ca^2+^, as compared with the treatment with its inactive isomer PP3 (Figure 2A–B). However, it had no effects on the Ca^2+^-induced disruption of the cortical F-actin stained with phalloidin-rhodamine B (Figure 2C–D).

We also analyzed the effects of the Src kinase inhibitor PP2 on the integrity of the cortical actin network in intact cells maintained in resting conditions or stimulated with 20 µM of the Ca^2+^ ionophore ionomycin during 10 s. Reportedly the cortical F-actin ring in chromaffin cells is visualized as an uninterrupted ring in resting conditions, but in the presence of a stimulus that increased cytosolic Ca^2+^ concentrations, such as ionomycin this F-actin ring is disrupted [5]. As shown in Figure 2E–H, this change in the cortical F-actin organization was also observed in cells treated with PP3 (10 µM for 20 min). PP2 did also not affect the cortical F-actin network under either resting or stimulated conditions.

Given that in ACCs SH2 and SH3 domains of Src-kinases mediate Src association to partners required for actin polymerization such as N-WASP and cortactin (see Figure S2), we next evaluated the impact of an acute disruption of these associations on the formation of F-actin. ACCs were permeabilized in the presence of 10 µM free Ca^2+^ and growing concentrations of chicken c-Src SH2- or SH3-GST fusion proteins. Amino acid sequences of chicken and bovine c-Src SH2 or SH3 domains are 100% identical, while sequence homology between chicken c-Src SH2 or SH3 domain and the respective domain of bovine Fyn or c-Yes are between 71 and 79% identical (see Table S1).

**Figure 3:**
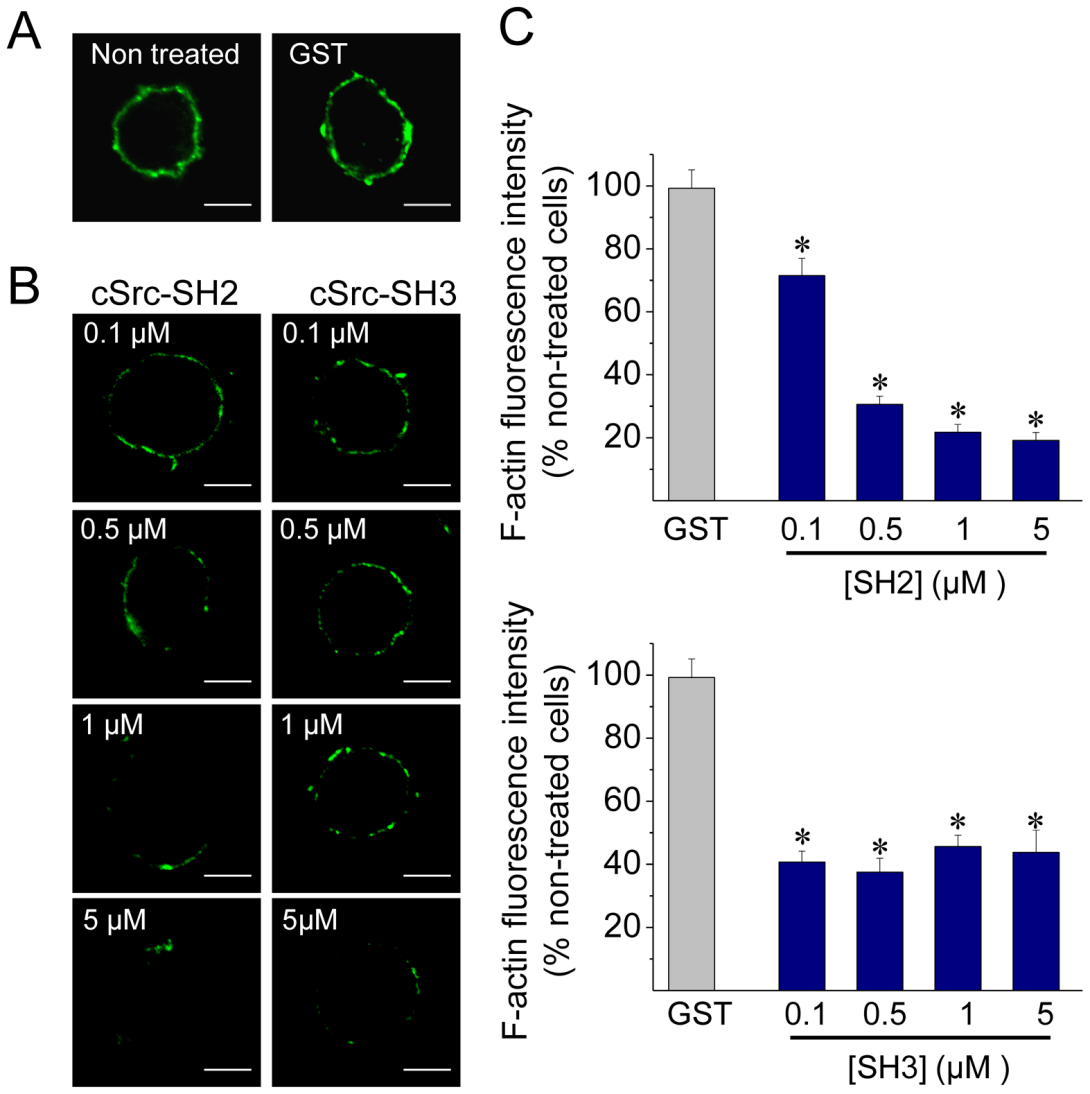
Disruption of c-Src SH2- or SH3-dependent associations inhibits de novo cortical actin assembly. ACCs were permeabilized in the presence of Alexa Fluor 488 G-actin, 10 µM free Ca^2+^ and 5 µM GST alone or growing concentrations of cSrc SH2-GST (SH2) or cSrc SH3-GST (SH3). (**A–B**) Representative images showing the new formation of cortical actin in cells permeabilized without peptides (non-treated) and in the presence of GST (A), or with different amounts of SH2 (B, left panel) or SH3 (B, right panel). Scale bar  = 10 µm. (**C**) Quantification demonstrates that 0.1 µM of c-Src SH2 or SH3 domain is sufficient to significantly disrupt Ca^2+^-dependent actin polymerization in ACCs. Data are means ± SEM of fluorescence intensity from at least 10 cells per each condition. *p<0.05 compared to GST.

As shown in Figures 3A–B, 0.1 µM of the c-Src SH2 or SH3 domain was able to significantly disrupt the new formation of actin filaments as compared with 5 µM GST alone. At such concentration, the inhibitory effect of the SH3 domain was larger than that of the SH2 domain (59.3% of inhibition with the SH3 domain v/s 28.5% of inhibition with the SH2 domain). These data indicate that Src kinase associations via SH2 or SH3 domain play an important role in promoting actin polymerization in ACCs.

### Src kinases regulate fusion pore expansion and the kinetics of exocytotic events in chromaffin cells

Since cortical actin actively participates during exocytosis adjusting the characteristics of the release events in neuroendocrine cells [5]–[6], [8] we investigated how Src kinase function influences exocytosis. The release of catecholamines was measured using amperometry with carbon fiber microelectrodes, a technique that has a good time resolution, and therefore yields information of the different steps of exocytosis [6], [24]. Amperometric spike parameters such as spike amplitude (Imax), spike charge (Q), spike half-width (t_1/2_) and rise time (tP) were analyzed. All these parameters provide information about the characteristics of the exocytotic events [5]. The number of exocytotic events and the current that precedes the spike, usually called foot, were also analyzed. Given that Src kinases influence both nicotinic receptor [29] and voltage-dependent Ca^2+^ channel activity [30], we induced exocytosis with the Ca^2+^ ionophore ionomycin (20 µM during 10 s). Src kinase activation was disrupted by the incubation of ACCs with 10 µM PP2 for 10 min before the stimulus.

**Figure 4:**
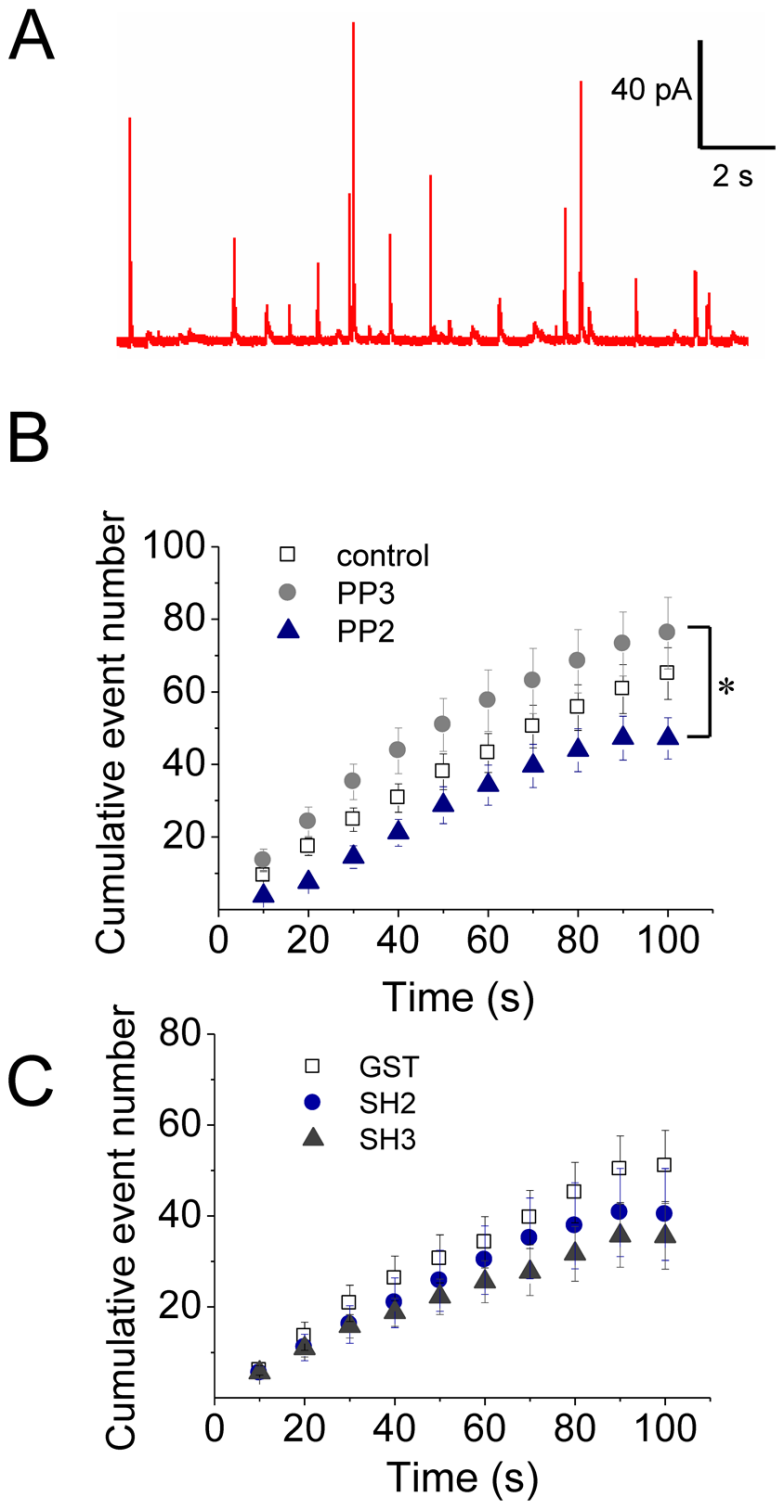
Src kinase inhibition reduces the number of exocytotic events. ACCs were incubated with the Src kinase inhibitor PP2 (10 µM) or its inactive isomer PP3 for 20 min, or injected with 5 µM GST, or c-Src SH2-GST (SH2) or cSrc SH3-GST (SH3) domain. Exocytosis was induced with the Ca^+2^ ionophore ionomycin (20 µM) and monitored by amperometry. (**A**) A representative amperometric trace from a non-treated cell (Control). (**B–C**) Cumulative histograms of the number of amperometric events from non-treated cells (control), or cells treated with PP2 or PP3, or injected with GST, SH2 or SH3. Data are means ± SEM from 12–20 cells. *p<0.05 compared with PP3.

**Figure 5:**
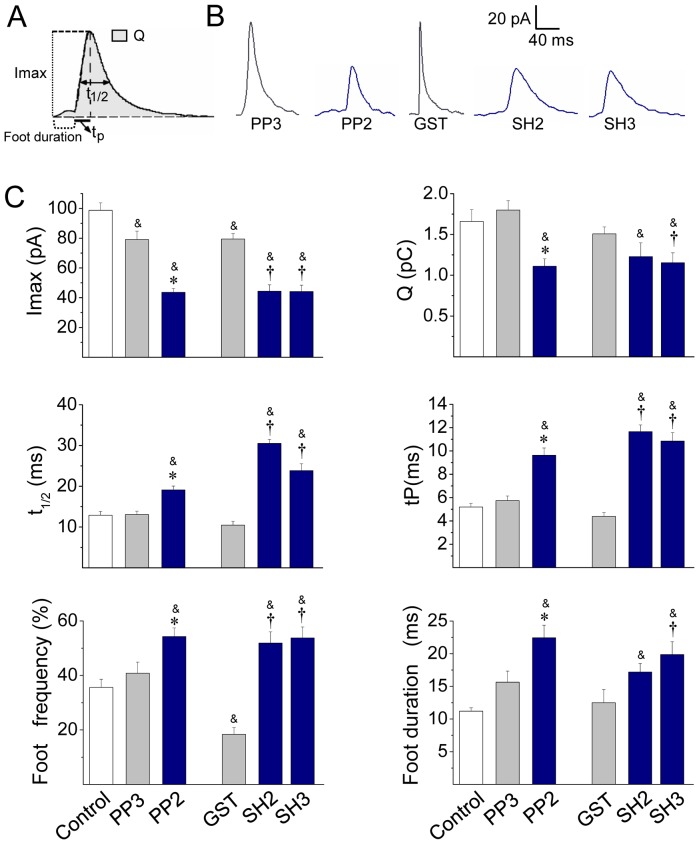
Src kinase inhibition slows down the fusion pore expansion. Exocytosis was induced with 20 µM ionomycin and monitored by amperometry. Cells were incubated with 10 µM PP2 or its inactive isomer PP3 for 20 min before the exocytosis induction. These agents were present during the recording. GST, c-Src SH2-GST (SH2) or c-Src SH3-GST (SH3) was injected 30 min before cell stimulation. (**A**) Scheme of an amperometric spike with the analyzed parameters: peak amplitude (Imax), quantal size (Q), half-width (t_1/2_), rise time (tP) and food duration. (**B**) Representative amperometric spikes from cells treated with PP3 or PP2, or injected with GST, SH2 or SH3. (**C**) Data show average values ± S.E.M. of Imax, Q, t_1/2_, tP, foot frequency and foot duration of amperometric events in control cells (n = 35) or cells treated with PP3 (n = 15), PP2 (n = 20) or injected with GST (n = 13), SH2 (n = 12), SH3 (n = 15). All amperometric parameter values correspond to the median values of the events from individual cells, which were subsequently averaged per treatment group. ^&^p<0.05 compared with control; *p<0.05 compared with PP3; ^†^p<0.05 compared with GST.

Figure 4A shows a typical amperometric recording in a non-treated control cell. In these cells a 10 s pulse of 20 µM ionomycin induced 65.0±7.1 amperometric spikes in 100 s (n = 35), in which average values of Imax, Q, t_1/2_ and tP were 98.7±5.0 pA, 1.7±0.1 pC, 12.9±0.9 ms and 5.2±0.3 ms, respectively. With the exception of the Imax that was reduced by 20%, the treatment with 10 µM PP3 did not modify other amperometric parameters (Figures 4 and 5). Conversely, Src kinase inhibition with 10 µM PP2 drastically changed the characteristics of the amperometric spikes (Figures 5B–C). Indeed, as compared with PP3, PP2 significantly reduced Imax and Q by 45% and 40% respectively, indicating that Src kinases control the amount of catecholamines released during individual events. We also found out that PP2 dramatically increased t_1/2_ and tP (Figure 5). Given that t_1/2_ reflects the duration of the exocytotic events and tP has been related with the kinetics of the fusion pore expansion [31]–[32], it is possible that the inhibition of Src kinases delays fusion pore expansion, giving rise to events with slow kinetics, and incomplete release. Therefore, we next analyzed the amperometric foot, which reflects the release of catecholamines through the initial fusion pore [33]. Here we observed that PP2 significantly increased the frequency of foot events, and prolonged the foot duration (Figure 5C), which reflects the stability of the fusion pore [7]. Conversely, PP2 did not influence the foot amplitude (Table S2) that correlates with the conductance of the fusion pore [34]. Thus, these latter findings confirm that Src kinases control fusion pore expansion. On the other hand, PP2 also significantly reduced the number of exocytotic events (Figure 4B). As we will discuss later this effect could be a consequence of the role of c-Src on the secretory vesicle trafficking [35]. Table S2 shows the values of the amperometric parameters in these latter conditions.

Src kinases usually bind to their partners via SH3 or SH2 domains [36]. Therefore, we studied the effects of the intracellular injection of the chicken c-Src SH2 or SH3 domain fused to GST on the characteristics of the exocytotic events. These fusion proteins were injected 30 min before the stimulus at a concentration of 5 µM, and their effects were compared with the injection of GST alone. As compared with non-injected cells (control), the injection of GST alone reduced Imax by 20% and the frequency of foot signals by 48% without affecting any other amperometric parameters (Table S2). Conversely, the injection of the c-Src SH2 or SH3 domain reduced Imax values by 55% and increased over twice t_1/2_ and tP, as compared with non-injected or GST-injected cells (Figure 5C). Also in comparison with GST, neither c-Src SH2 nor SH3 affected the number of exocytotic events (Figure 4B), but they increased the frequency of foot signals (Figure 5C). On the other hand, the injection of the c-Src-SH3, but not of the cSrc-SH2, significantly reduced Q and prolonged foot duration (Figure 5C). Table S2 shows the values of the amperometric parameters in these latter conditions.

**Figure 6:**
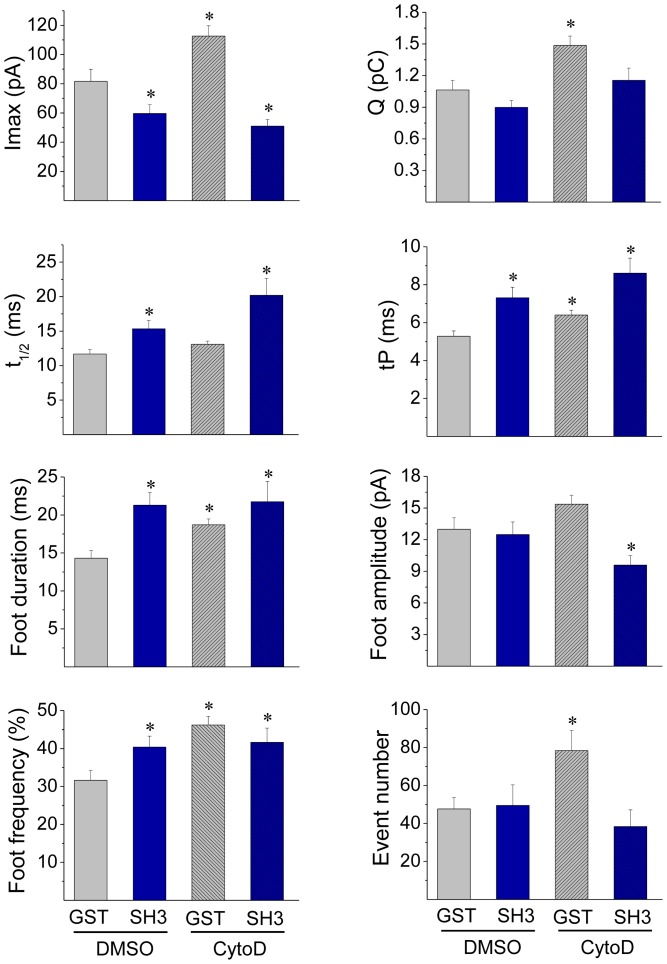
Cytochalasin D does not affect the quantal release in cells injected with the cSrc SH3 domain. ACCs were injected with 5 µM GST or c-Src SH3-GST (SH3), and 20 min later incubated with 4 µM cytochalasin D (CytoD) or its vehicle DMSO for 10 min at 37°C. Then, exocytosis was induced with 20 µM ionomycin and monitored by amperometry. Data show average values ± S.E.M. of Imax, Q, t_1/2_, tP, foot duration, foot amplitude, foot frequency and number of events during the recording from cells injected with GST and treated with DMSO (n = 15) or CytoD (n = 16) or injected with SH3 treated with DMSO (n = 24) or CytoD (n = 14). All amperometric parameter values correspond to the median values of the events from individual cells, which were subsequently averaged per treatment group. *p<0.05 compared with cells injected with GST and treated with DMSO. Note that SH3 and CytoD have common effects in tP, foot duration and foot frequency, but show dissimilar effects on Imax and Q. In the latter parameters, the effects of c-Src-SH3 prevail over those of CytoD.

In order to determine whether c-Src-SH3 regulates exocytosis through an actin-mediated pathway, we analyzed the effects of the injection of c-Src-SH3 in the presence of the actin-disrupting drug Cytochalasine D (CytoD). As shown in Figure 6, in cells injected with GST alone, CytoD significantly increased tP, foot duration and foot frequency. These same effects were observed in cells injected with cSrc-SH3 and treated with DMSO or CytoD. Regarding the fusion pore conductance, neither the injection of cSrc-SH3 nor the incubation with CytoD affected the foot amplitude; however, the combination of both treatments significantly decreased this foot parameter. On the other hand, contrary to what we observed with the injection of cSrc-SH3, CytoD significantly increased Imax and Q (Fig. 6), but such effects were not observed in cells injected with cSrc-SH3 and treated with CytoD. In the latter condition Imax importantly decreased, while Q was not affected. Table S3 shows the values of the amperometric parameters in these latter conditions.

**Figure 7:**
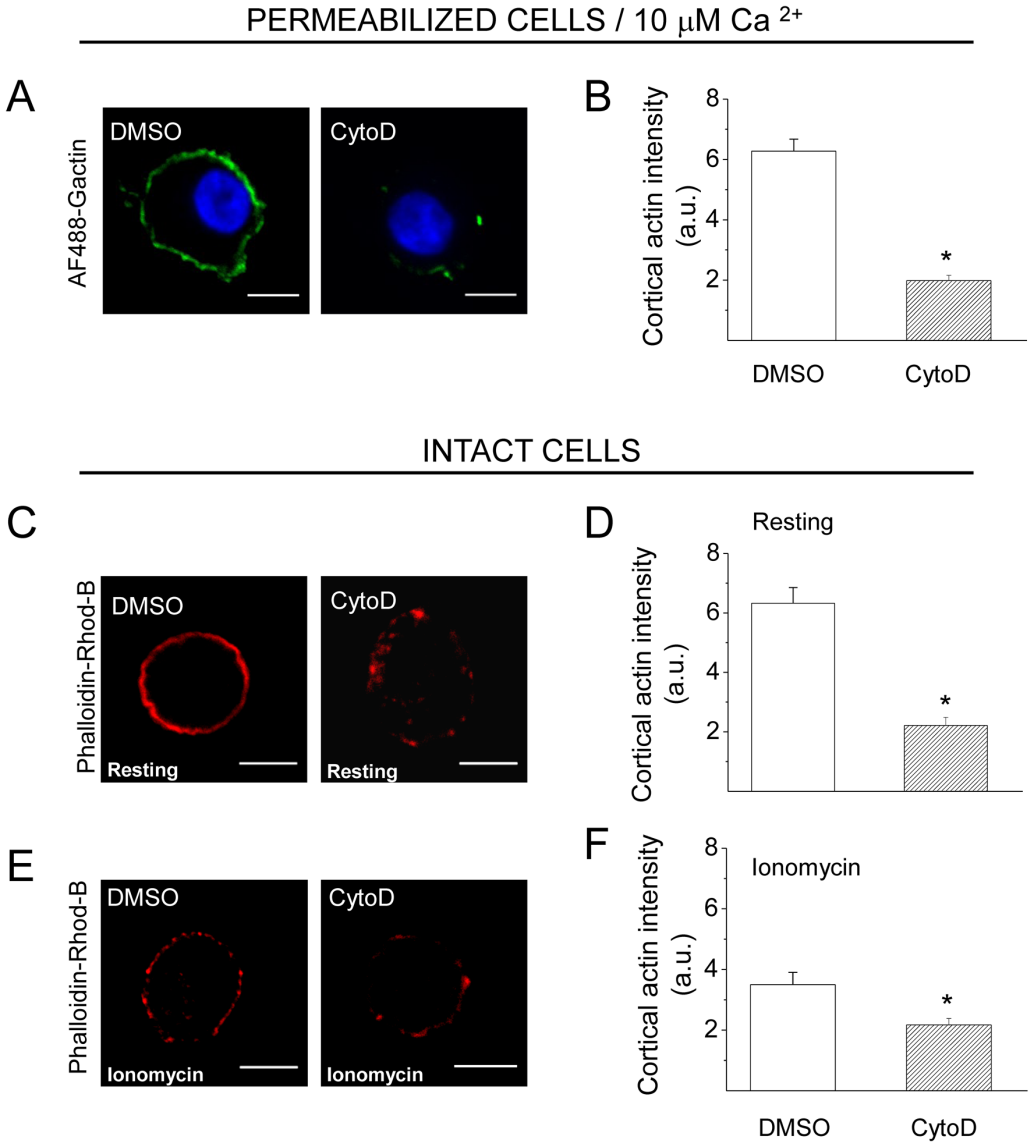
Cytochalasin D inhibits the new formation of actin filaments and disturbs the subplasmalemmal actin network in ACCs. (**A–B**) Cultured chromaffin cells were permeabilized in the presence of 0.3 µM AF488-G-actin, 10 µM free Ca^2+^ and 4 µM of CytoD or the vehicle DMSO. Note that CytoD treatment drastically inhibited the *de novo* actin polymerization. (**C–F**) Intact cells were incubated for 10 minutes with 4 µM CytoD, or the vehicle DMSO, at 37°C, then maintained resting conditions (C–D) or stimulated with 20 µM ionomycin (E–F), fixed, stained with phalloidin-rhodamine B and visualized by confocal microscopy. Note that Cyto D significantly reduced the cortical F-actin signal in both, resting and stimulated cells. A, C and E show representative confocal images for each condition. Scale bar  = 10 µm. B, D and F correspond to quantification of the cortical actin fluorescence intensity, where data are means ± SEM from at least 15 cells per each condition. *p<0.05 compared to DMSO.

**Figure 8:**
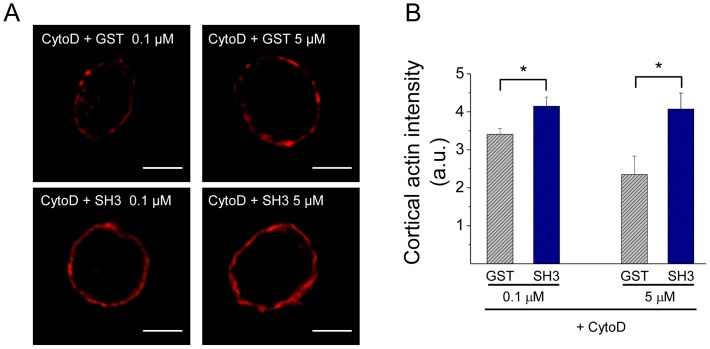
c-Src-SH3 diminishes the F-actin disruption induced by cytochalasin D. ACCs were digitonin-permeabilized for 6 minutes in the presence of 10 µM free Ca^2+^, 4 µM cytochalasin D (CytoD) and GST or c-Src-SH3-GST (SH3) at 0.1 or 5 µM. Then cells were fixed and stained with 1 µM of phalloidin-rhodamine B. (**A**) Representative images of each experimental condition. Scale bar  = 10 µm. (**B**) Quantification of the cortical actin intensity fluorescence. Data are means ± SEM for at least 18 cells from 3 different cultures per each condition. Note a significantly higher cortical F-actin intensity in cells treated with CytoD in the presence of c-Src-SH3, at either 0.1 or 5 µM, as compared with cells treated with CytoD in the presence of GST (*p<0.05).

The disrupting effects of CytoD on the formation of new actin filaments and on the organization of the actin network in intact cells are shown in Figure 7. Similar to that observed with Src kinase inhibition, CytoD suppressed the new F-actin formation (Figure 7A–B). However, contrary to what we observed with Src inhibition, CytoD disrupted the cortical actin network in resting conditions (Figure 7C–D) and induced a further F-actin disruption in ionomycin-stimulated cells (Figure 7E–F). Therefore, we analyzed how the combined treatment of CytoD plus cSrc-SH3 affects the cortical actin network in the presence of high Ca^2+^ concentrations. In order to adjust cSrc-SH3 concentrations, these experiments were performed in cells permeabilized in the presence of 10 µM free Ca^2+^. In this condition we observed that cSrc-SH3 at both concentrations (0.1 and 5 µM), significantly diminished the F-actin disruption observed in the presence of CytoD (Figure 8).

### Inhibition of N-WASP suppresses cortical actin polymerization and reduces the quantal release

**Figure 9:**
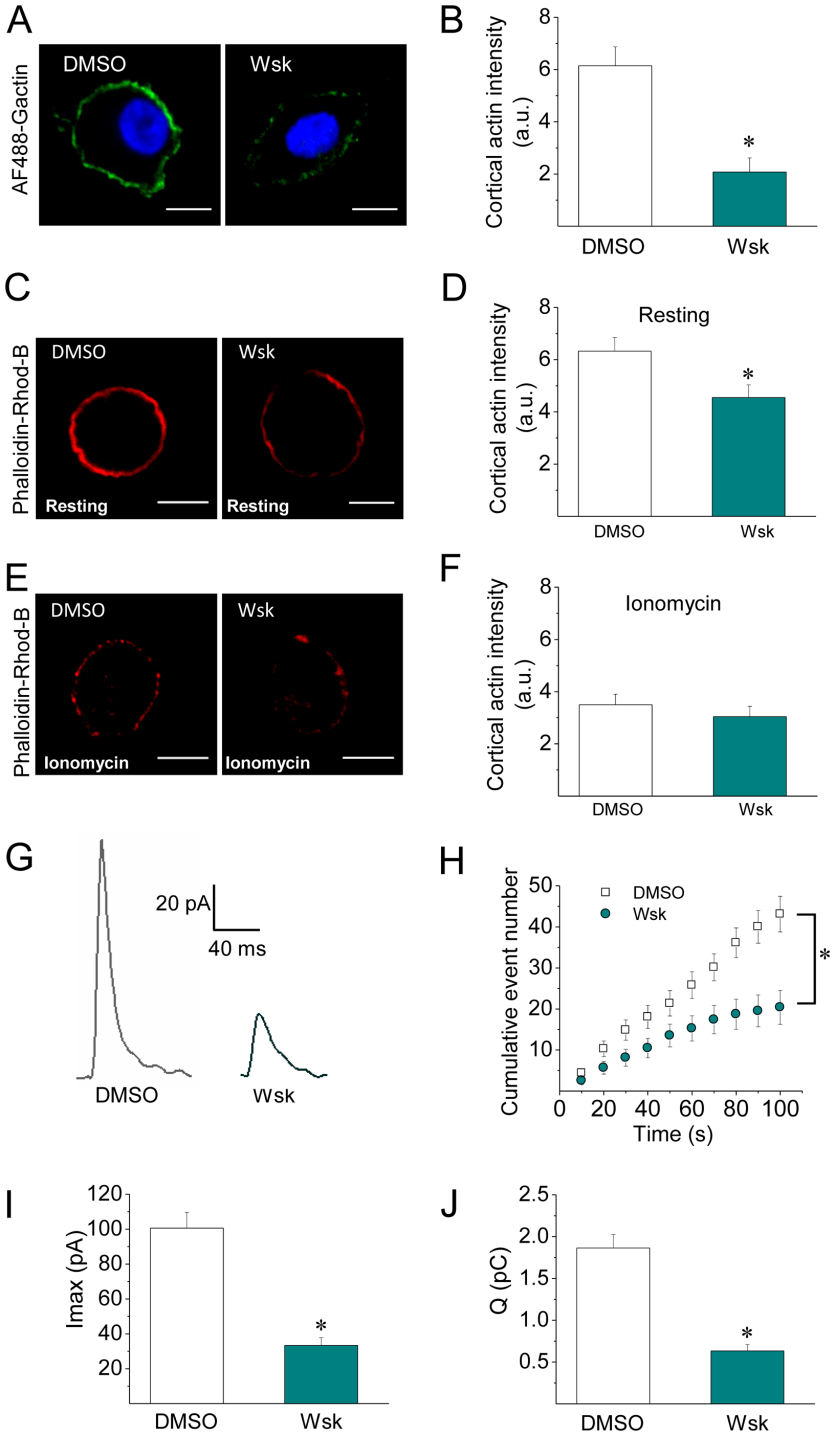
Inhibition of N-WASP activation suppresses de novo actin polymerization and reduces the quantal release of transmitters. (**A–B**) ACCs were permeabilized in the presence of 10 µM free Ca^2+^ and 5 µM of the N-WASP inhibitor wiskostatin (Wsk), or the vehicle DMSO. The new F-actin formation was evaluated in the presence of 0.3 µM of AF488-G-actin. Note that Wsk treatment significantly disrupted the new F-actin polymerization. (**C–F**) Cultured ACC were incubated with the N-WASP inhibitor Wsk for 5 minutes at 37°C, then maintained in resting conditions (C–D) or stimulated with 20 µM ionomycin (E–F), fixed and stained with phalloidin-rhodamine B for confocal visualization. Note that Wsk slightly disturbs the cortical actin ring in resting cells, but it did not induce an additional F-actin disruption in ionomycin treated cells. A, C and E show representative confocal images for each condition. Scale bar  = 10 µm. B, D and F correspond to quantification of the cortical actin fluorescence intensity, where data are means ± SEM from at least 11 cells per each condition. *p<0.05 compared to DMSO. (**G–J**) Cultured ACCs were incubated with 5 µM Wsk for 5 min prior to the exocytosis induction, then this agent was kept during the amperometric recording. Representative amperometric spikes are shown in panel G. Data show average values ± S.E.M. of the number of cumulative exocytotic events (H), Imax (I) or Q (J) from cells treated with DMSO (n = 29) or Wsk (n = 15). The amperometric parameter values correspond to the median values of the events from individual cells, which were subsequently averaged per treatment group. Note that the inhibition of N-WASP activation with Wsk reduced the number of exocytotic events (D), and decreased the amplitude (E) and quantal size (F).*p<0.05 compared to DMSO.

Given that N-WASP contributes to the formation of actin filaments during exocytosis in chromaffin cells [3], we evaluated the effect of wiskostatin (Wsk), an agent that prevents N-WASP activation by stabilizing its autoinhibited conformation [37]. As shown in Figure 9A–B, 5 µM of Wsk efficiently inhibited the *de novo* cortical actin polymerization in cells permeabilized in the presence of 10 µM free Ca^2+^. Wsk also disturbed the subplasmalemmal actin ring under the resting condition, reducing the F-actin labelling by 27% (Figure 9C–D), but it did not induce any additional disturbance in the cortical actin in ionomycin stimulated cells (Figure 9E–F).

We also evaluated the effects of 5 µM Wsk on the characteristics of exocytosis and found out that Wsk significantly reduced the number of individual exocytotic events (Figure 9H) and reduced Imax and Q by almost 70% (Figure 9I–J). However, t_1/2_ and tP values were not affected by the treatment with the N-WASP inhibitor (Table S4). As shown in Table S4, only 26.8±0.5 of spikes exhibited foot signals, which had significantly smaller amplitude, as compared with cells treated with the vehicle DMSO (Table S4).

## Discussion

The cortical actin network constitutes a dynamic structure that controls and directs the motion of secretory vesicles to the plasma membrane [38]. As demonstrated here, both actin polymerization and depolymerization are induced by Ca^2+^ in ACCs (Figure 1). Pioneering studies of Trifaró and collaborators [1] showed that high cytosolic Ca^2+^ levels activate the actin filament-severing protein scinderin, inducing disassembly of the cortical actin network and allowing secretory vesicles to reach the plasma membrane [39]. As later reported, a localized F-actin assembly at the exocytotic sites appears to take place in response to stimulation of neuroendocrine cells [3]. However, how a stimulation-induced increase in the cytosolic Ca^2+^ levels activates F-actin assembly is still unclear. As it is discussed here, one possibility is that Src kinases orchestrate this Ca^2+^-dependent actin polymerization and thereby modulate different stages of exocytosis.

In the present paper, we found that in ACCs Src kinases regulate actin polymerization (Figure 2) and the fusion pore expansion (Figure 5). These Src kinase actions seem to be dependent on Src kinase catalytic activity as well as on the associations to other proteins through its SH2 and SH3 domains (Figure 3–5). As shown here, these two domains are also involved in the association of c-Src to the NPFs N-WASP and cortactin in ACCs (Figures S2). Both N-WASP and cortactin are known Src kinase substrates [11], [40]. Furthermore, Src kinases regulate N-WASP/cortactin interaction [25], [41], which acts synergistically during actin polymerization [42]–[44].

**Figure 10:**
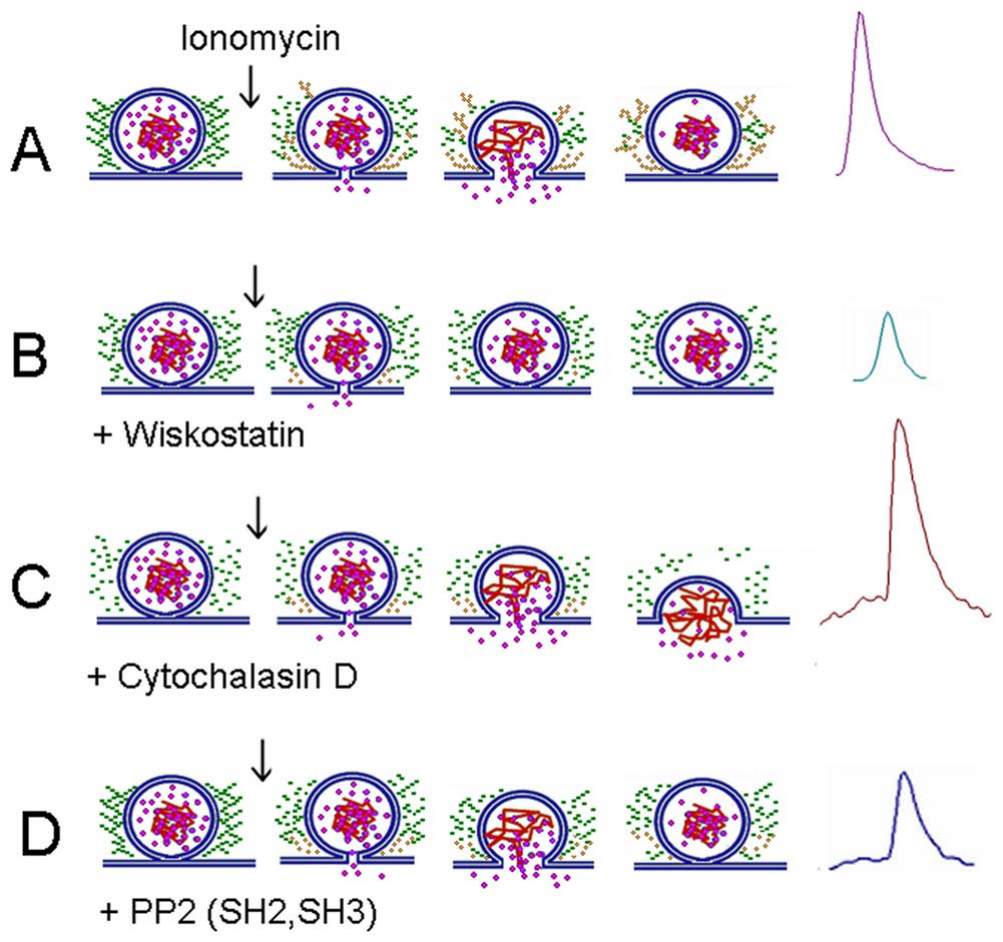
Influence of the different treatments on actin dynamics. (**A**) A stimulus that induces an increase in the cytosolic Ca^2+^ hastens SNARE-mediated fusion of secretory vesicles with the plasma membrane, but it also promotes both disruption of the preexisting actin network (green rosary beads) and the formation of new actin filaments (yellow rosary beads). This actin dynamics appears to favor the expansion of the fusion pore, but also prevents the collapse of the vesicle in the plasma membrane. This mechanism allows the fast release of soluble catecholamines (see the purple spike at the right). (**B**) The N-WASP inhibitor wiskostatin (Wks) strongly inhibits the new actin polymerization, while it slightly disturbs the preexisting cortical F-actin network (Figure 9). Such effect could be due to a reduction of the slow-rate of actin polymerization/depolymerization in resting conditions. Wsk also gives rise to incomplete release events (see the small green spike), suggesting that the lack of actin polymerization hinders the expansion of the fusion pore. Wsk could also perturb membrane transport, decreasing cellular ATP levels and Ca^2+^ entry [52]. These effects could also contribute to the failure of the fusion pore expansion. (**C**) Cytochalasin D (CytoD) greatly disrupts the preexisting F-actin network, as well as the new F-actin formation (Figure 7). CytoD also slows down the fusion pore expansion and increases the quantal size of the release events (see the big brown spike with a long foot). Probably the loss of actin meshwork favors the collapse of the vesicle in the plasma membrane, as previously proposed by Doreian *et al.* [8]. (**D**) The inhibition of Src kinases with PP2 suppresses the *de novo* F-actin formation, but it does not disturb the pre-existing cortical actin network (Fig. 2). PP2 also slows down the fusion pore expansion, and reduces the quantal size of the release events (note the small blue spike with a long foot). Similar effects were observed with the microinjections of cSrc-SH2 or -SH3 domains. These findings support the idea that the new F-actin formation favors the expansion of the fusion pore.

Probably as a consequence of its actions on actin polymerization, Src kinases regulate the fusion pore expansion. In fact, the inhibition of Src kinase activity with PP2, or the disruption of its association to other proteins through its SH3 or SH2 domain has some effects that are similar to those produced by the actin-disrupting drug CytoD. Both treatments inhibited *de novo* actin polymerization (Figures 2, 3 and 7), and slowed down fusion pore expansion: they increased tP, foot duration and foot frequency (Fig. 5 and 6; see also [6]). However, differently to that observed with CytoD, Src kinase inhibition did not disturb the cortical F-actin organization under resting or stimulated conditions (compare Figure 2E–H with Figures 7C–F), neither increased the amperometric parameters Imax and Q (Figures 5 and 6). These findings suggest to us that the *de novo* actin polymerization and the preexisting F-actin network regulate different steps of exocytosis; while the new F-actin formation appears to promote the expansion of the initial fusion pore, the preexisting F-actin would control a later step of the exocytosis. Corey Smith and collaborators [8] proposed that the disruption of the cortical F-actin network (observed in cells subjected to a strong stimulus or in the presence of CytoD) favors the full collapse of the secretory vesicles into the plasma membrane, while an uninterrupted actin network (that is maintained in cells subjected to low frequency stimulation) would prevent the vesicle collapse and thereby favoring a kiss-and-run mechanism [8]. Since CytoD does not affect the vesicle volume in ACCs [5], [6], Smith's hypothesis could explain the increase in the quantal size induced by CytoD observed here (Figure 6) and also in our previous work [8]. This idea is depicted in the Figure 10C.

The combined treatment of CytoD with the cSrc-SH3 domain also supports the idea that cSrc kinase and the de novo actin polymerization regulate the expansion of the initial fusion pore, and not later steps of exocytosis. As shown in Figures 6 and 9, the cSrc-SH3 domain diminished the disruption of the cortical actin network induced by CytoD, and prevented the increase of Imax and Q also induced by this actin-disrupting agent. How cSrc-SH3 domain prevents the actin disruption is not clear to us at this moment. One possibility is that this action is mediated by the GTPase dynamin. In fact, dynamin is a Src substrate involved in regulating actin dynamics in several cellular processes [45]–[46]. Furthermore, Src kinase regulates the association of dynamin to cortactin [47] and the complex dynamin/cortactin stabilizes F-actin bundles [48]. In ACCs, c-Src binds dynamin via its SH3 domain (see Figure S2C) and as we previously demonstrated dynamin regulates the organization of the cortical actin [6].

In order to understand better the role of the new F-actin formation on the dynamics of the fusion pore, we analyzed the effects of Wsk on the cortical actin dynamics and the amperometric spikes. As observed with CytoD, this N-WASP inhibitor suppressed the Ca^2+^-dependent actin polymerization (Figure 9A–B). However, it slightly disturbed the actin network in resting condition (Figure 9C–D), and did not affect the ionomycin-induced cortical F-actin disruption (Figure 9E–F). Interestingly, the effects of Wsk on the exocytosis were totally different to those of CytoD: Wsk significantly reduced Imax and Q. Similar effects were observed by Corey Smith and collaborators using Wsk during high frequency stimulation [49]. We interpret these effects as an inhibition of the fusion pore expansion that gives rise to “stand-alone foot” events [34], [50]–[51]. In this type of event only a small fraction of the intravesicular catecholamines is released [34], [51].

Based on the similar effects of Wsk and PP2, as well as the effects of cSrc-SH3, on the spike amplitude and quantal size (compare Figure 5 and Figure 9), we propose that N-WASP and Src kinases operate through a common mechanism in exocytosis, which relies on new actin polymerization (see our model in Figure 10). This mechanism seems to be perturbed differently by Wsk or PP2. While Wsk appears to prevent the expansion of the initial fusion pore, giving rise to “stand-alone foot” events, PP2 only decelerates the pore expansion, generating amperometric events with a long foot signal, slow rise time and small quantal size. They affect the fusion pore dynamics differently, probably because N-WASP is directly involved in actin nucleation, while Src kinases regulate actin dynamics by acting on different substrates and modulating their associations. Besides this, Wsk reportedly perturbs membrane transport, decreasing cellular ATP levels [52] and Ca^2+^ influx [49]. These actions of Wsk could further contribute to the failure of the fusion pore expansion, and also to the dramatic reduction in the number of exocytotic events. Indeed, these two types of processes are highly dependent on Ca^2+^ levels [53].

However, it must be taken into account that the fusion pore expansion is not the only step of the secretory pathway that could be affected by the perturbation of the actin dynamics. Since actin dynamics is critical in maintaining a proper Golgi-complex architecture and function [54], we cannot rule out that an actin-disrupting treatment influences early stages of the secretory pathway and thus modifies vesicle size, vesicle content and/or number of exocytotic events.

Regarding the mechanism by which the different treatments affect the amount of exocytotic events, our data show that Wsk and PP2 decreased the number of amperometric spikes by 52% and 38%, respectively, while CytoD increased it by 38% (see Tables S2, S3 and S4). As aforementioned, the preexisting cortical actin network constitutes a barrier that limits the access of secretory vesicles to plasma membrane [1], therefore its disruption with CytoD increases the number of exocytotic events. On the other hand, F-actin appears to form trails that direct the motion of vesicles to the exocytotic sites [4]. Src kinase function could modulate this last process since it phosphorylates the small insert isoform of myosin VI making it able to bind secretory vesicles in a Ca^2+^-dependent manner, and then recruit them to the cortical actin network [35].

Therefore, the preexisting cortical actin network and the newly formed F-actin appear to regulate differently the access of secretory vesicles to the plasma membrane, the expansion of the fusion pore and the mode of exocytosis. Both the disruption of the preexisting actin and the formation of new actin filaments rely on cytosolic Ca^2+^ levels, as well as on the concerted action of proteins, among them Src kinases that play a pivotal role in regulating Ca^2+^-dependent actin polymerization. Figure 10 depicts our proposed model of how this mechanism works.

## Supporting Information

Figure S1 **Cell permeabilization does not affect Src kinases/cortactin ratio.** In order to determine if Src kinases are lost during permeabilization with digitonin (20 µM for 6 min), we compared the protein levels of Src kinases (60 kDa) with the levels of a protein with a higher molecular weight, cortactin (82 kDa) in total lysates obtained from intact cells (I.C.) and digitonin-permeabilized cells (P.C.). The upper panel shows a representative western blot. The lower panel shows the densitometric analysis of 4 different experiments. The bars represent the average of Src kinases/cortactin ratio. We did not observed differences between the two treatments.
(TIF)

Figure S2 **c-Src partners in adrenal chromaffin cells.** (**A–C**) ACC extracts were subjected to pull-down assays using GST, N-WASP PRD-GST (N-W-PRD) (A), c-Src SH2-GST (B) or cSrc SH3-GST (C). Bound proteins were evaluated by immunoblotting using specific antibodies against Src-kinases (A), cortactin (B) dynamin (C) or GST. Note that N-W-PRD pulled-down Src kinases from ACC (A). c-Src-SH2 (B) and c-Src-SH3 (C) efficiently pulled-down cortactin and dynamin from ACC extracts, respectively.
(TIF)

Table S1 **Homology of the amino acid sequences of chicken c-Src SH2 or SH3 domains with the same domains of bovine c-Src, Fyn and c-Yes.** For amino acid sequence comparison see: chicken c-Src: Genbank V00402.1, bovine c-Src: UniProt E1BIM8_BOVIN, bovine Fyn: NCBI NM_001077972.1, bovine c-Yes NCBI NM_001101060.1.
(DOC)

Table S2 **Amperometric parameters of exocytotic events induced by 20 µM ionomycin in cells treated with the Src kinase inhibitor PP2 or its inactive analogue PP3, or injected with c-Src SH2-GST, c-Src SH3-GST or GST alone.** Exocytosis was induced with 20 µM ionomycin and monitored by amperometry. Cells were incubated with 20 µM PP2 or its inactive analog PP3 20 min before the exocytosis induction. These agents were kept during the recording. GST, c-Src SH2-GST or c-Src SH3-GST were injected 30 min before cell stimulation. Control corresponds to non-treated cells. Data are means ± SEM of averages. &p<0.05 compared with control; *p<0.05 compared with cells treated with PP3; †p<0.05 compared with cells injected with GST.
(DOC)

Table S3 **Amperometric parameters of exocytotic events cells injected with c-Src SH3-GST and treated with cytochalasin D.** Exocytosis was induced with 20 µM ionomycin and monitored by amperometry. Cells injected with 5 µM GST or c-Src SH3-GST (SH3) were incubated with 4 µM cytochalasin D (CytoD) or its vehicle DMSO during 10 min at 37°C before the exocytosis induction and kept during the recording. Data are means ± SEM of averages. *p<0.05 compared with cells injected with GST and treated with DMSO.
(DOC)

Table S4 **Amperometric parameters of exocytotic events induced by 20 µM ionomycin in cells treated with wiskostatin.** Exocytosis was induced with 20 µM ionomycin and monitored by amperometry. Cells were incubated with 5 µM of the N-WASP inhibitor wiskostatin (Wsk) or the vehicle DMSO. Data are means ± SEM of averages. *p<0.05 compared with cells treated with DMSO.
(DOC)

## References

[1] Trifaró JM, Rosé SD, Marcu MG (2000) Scinderin, a Ca^2+^-dependent actin filament severing protein that controls cortical actin network dynamics during secretion. Neurochem Res 25: 133–144. 10685613 10.1023/a:1007503919265

[2] Malacombe M, Bader MF, Gasman S (2006) Exocytosis in neuroendocrine cells: new tasks for actin. Biochim Biophys Acta 1763: 1175–1183. 17034880 10.1016/j.bbamcr.2006.09.004

[3] Gasman S, Chasserot-Golaz S, Malacombe M, Way M, Bader MF (2004) Regulated Exocytosis in Neuroendocrine Cells: A Role for Subplasmalemmal Cdc42/N-WASP-induced Actin Filaments. Mol Biol Cell 15: 520–531. 14617808 10.1091/mbc.E03-06-0402 PMC329227

[4] Giner D, Neco P, Francés Mdel M, López I, Viniegra S, et al (2005) Real-time dynamics of the F-actin cytoskeleton during secretion from chromaffin cells. J Cell Sci 118: 2871–2880. 15976446 10.1242/jcs.02419

[5] Berberian K, Torres AJ, Fang Q, Kisler K, Lindau M (2009) F-actin and myosin II accelerate catecholamine release from chromaffin granules. J Neurosci 29: 863–870. 19158310 10.1523/JNEUROSCI.2818-08.2009 PMC2768403

[6] González-Jamett AM, Momboisse F, Guerra MJ, Ory S, Báez-Matus X, et al (2013) Dynamin-2 regulates fusion pore expansion and quantal release through a mechanism that involves actin dynamics in neuroendocrine chromaffin cells. PLoS One 8: e70638. 23940613 10.1371/journal.pone.0070638 PMC3734226

[7] Lindau M, Alvarez de Toledo G (2003) The fusion pore. Biochim Biophys Acta 1641: 167–173. 12914957 10.1016/s0167-4889(03)00085-5

[8] Doreian BW, Fulop TG, Smith CB (2008) Myosin II activation and actin reorganization regulate the mode of quantal exocytosis in mouse adrenal chromaffin cells. J Neurosci 28: 4470–4478. 18434525 10.1523/JNEUROSCI.0008-08.2008 PMC2745116

[9] Tehrani S, Tomasevic N, Weed S, Sakowicz R, Cooper JA (2007) Src phosphorylation of cortactin enhances actin assembly. Proc Natl Acad Sci U S A 104: 11933–11938. 17606906 10.1073/pnas.0701077104 PMC1924558

[10] Singh VP, McNiven MA (2008) Src-mediated cortactin phosphorylation regulates actin localization and injurious blebbing in acinar cells. Mol Biol Cell 19: 2339–23347. 18353971 10.1091/mbc.E07-11-1130 PMC2366849

[11] Torres E, Rosen MK (2006) Protein-tyrosine kinase and GTPase signals cooperate to phosphorylate and activate Wiskott-Aldrich syndrome protein (WASP)/neuronal WASP. J Biol Chem 28: 3513–3520. 10.1074/jbc.M509416200 16293614

[12] Yoo Y, Ho HJ, Wang C, Guan JL (2010) Tyrosine phosphorylation of cofilin at Y68 by v-Src leads to its degradation through ubiquitin-proteasome pathway. Oncogene 29: 263–272. 19802004 10.1038/onc.2009.319 PMC2806939

[13] De Corte V, Demol H, Goethals M, Van Damme J, Gettemans J, Vandekerckhove J (1999) Identification of Tyr438 as the major in vitro c-Src phosphorylation site in human gelsolin: a mass spectrometric approach. Protein Sci 8: 234–241. 10210201 10.1110/ps.8.1.234 PMC2144107

[14] Finkelstein M, Etkovitz N, Breitbart H (2010) Role and regulation of sperm gelsolin prior to fertilization. J Biol Chem 285: 39702–39709. 20937821 10.1074/jbc.M110.170951 PMC3000951

[15] Tatosyan AG, Mizenina OA (2000) Kinases of the Src family: structure and functions. Biochemistry (Mosc) 65: 49–58. 10702640

[16] Parsons SJ, Parsons JT (2004) Src family kinases, key regulators of signal transduction. Oncogene 23: 7906–7909. 15489908 10.1038/sj.onc.1208160

[17] Monteiro AN (2006) Involvement of the SH3 domain in Ca2+-mediated regulation of Src family kinases. Biochimie 88: 905–911. 16546311 10.1016/j.biochi.2006.01.013

[18] Zhao Y, Sudol M, Hanafusa H, Krueger J (1992) Increased Tyrosine Kinase Activity of c-Src During Calcium-Induced Keratinocyte Differentiation. Proc Natl Acad Sci U S A 89: 8298–8302. 1381508 10.1073/pnas.89.17.8298 PMC49905

[19] Allen CM, Ely CM, Juaneza MA, Parsons SJ (1996) Activation of Fyn tyrosine kinase upon secretagogue stimulation of bovine chromaffin cells. J Neurosci Res 44: 421–429. 8776663 10.1002/(SICI)1097-4547(19960601)44:5<421::AID-JNR2>3.0.CO;2-H

[20] Rusanescu G, Qi H, Thomas SM, Brugge JS, Halegoua S (1995) Calcium influx induces neurite growth through a Src-Ras signaling cassette. Neuron 15: 1415–1425. 8845164 10.1016/0896-6273(95)90019-5

[21] Wang SJ (2003) A role for Src kinase in the regulation of glutamate release from rat cerebrocortical nerve terminals. Neuroreport 14: 1519–1522. 12960777 10.1097/00001756-200308060-00024

[22] Zhang Z, Fan J, Ren Y, Zhou W, Yin G (2013) The release of glutamate from cortical neurons regulated by BDNF via the TrkB/Src/PLC-γ1 pathway. J Cell Biochem 114: 144–1451. 22886995 10.1002/jcb.24311

[23] Parsons SJ, Creutz CE (1986) p60c-src activity detected in the chromaffin granule membrane. Biochem Biophys Res Commun 134: 736–742. 3511908 10.1016/s0006-291x(86)80482-x

[24] Ardiles AO, González-Jamett AM, Maripillán J, Naranjo D, Caviedes P (2007) Calcium channel subtypes differentially regulate fusion pore stability and expansion. J Neurochem 103: 1574–1581. 17760862 10.1111/j.1471-4159.2007.04871.x

[25] Martinez-Quiles N, Ho HY, Kirschner MW, Ramesh N, Geha RS (2004) Erk/Src phosphorylation of cortactin acts as a switch on-switch off mechanism that controls its ability to activate N-WASP. Molecular Cell Biol 24: 5269–5280. 10.1128/MCB.24.12.5269-5280.2004 PMC419870 15169891

[26] Ardiles AO, Maripillán J, Lagos VL, Toro R, Mora IG, et al (2006) A rapid exocytosis mode in chromaffin cells with a neuronal phenotype. J Neurochem 99: 29–41. 16889641 10.1111/j.1471-4159.2006.04080.x

[27] García-Palomero E, Montiel C, Herrero CJ, García AG, Alvarez RM, et al (2000) Multiple calcium pathways induce the expression of SNAP-25 protein in chromaffin cells. J Neurochem 74: 1049–10458. 10693936 10.1046/j.1471-4159.2000.0741049.x

[28] Vitale N, Mukai H, Rouot B, Thiersé D, Aunis D, et al (1993) Exocytosis in chromaffin cells. Possible involvement of the heterotrimeric GTP-binding protein Go. J Biol Chem 268: 14715–14723. 7686903

[29] Wang K, Hackett J, Cox M, Hoek M, Lindstrom J, et al (2004) Regulation of the Neuronal Nicotinic Acetylcholine Receptor by Src Family Tyrosine Kinases. J Biol Chem 279: 8779–8786. 14679211 10.1074/jbc.M309652200

[30] Wijetunge S, Lymn JS, Hughes AD (2000) Effects of protein tyrosine kinase inhibitors on voltage-operated calcium channel currents in vascular smooth muscle cells and pp60(c-src) kinase activity. Br J Pharmacol 129: 1347–1354. 10742290 10.1038/sj.bjp.0703186 PMC1571969

[31] Schroeder TJ, Borges R, Finnegan JM, Pihel K, Amatore C, et al (1996) Temporally resolved, independent stages of individual exocytotic secretion events. Biophys J 70: 1061–1068. 8789125 10.1016/S0006-3495(96)79652-2 PMC1225008

[32] Neco P, Fernández-Peruchena C, Navas S, Gutiérrez LM, de Toledo GA, et al (2008) Myosin II contributes to fusion pore expansion during exocytosis. J Biol Chem 283: 10949–10957. 18283106 10.1074/jbc.M709058200

[33] Chow RH, Von Rüden L, Neher E (1992) Delay in vesicle fusion revealed by electrochemical monitoring of single secretory events in adrenal chromaffin cells. Nature 356: 60–63. 1538782 10.1038/356060a0

[34] Albillos A, Dernick G, Horstmann H, Almers W, Alvarez De Toledo G, et al (1997) The exocytotic event in chromaffin cells revealed by patch amperometry. Nature 389: 509–512. 9333242 10.1038/39081

[35] Tomatis VM, Papadopulos A, Malintan NT, Martin S, Wallis T, et al (2013) Myosin VI small insert isoform maintains exocytosis by tethering secretory granules to the cortical actin. J Cell Biol 200: 301–320. 23382463 10.1083/jcb.201204092 PMC3563687

[36] Engen JR, Wales TE, Hochrein JM, Meyn MA, Banu Ozkan S, et al (2008) Structure and dynamic regulation of Src-family kinases. Cell Mol Life Sci 65: 3058–3073. 18563293 10.1007/s00018-008-8122-2 PMC9357288

[37] Peterson JR, Bickford LC, Morgan D, Kim AS, Ouerfelli O, et al (2004) Chemical inhibition of N-WASP by stabilization of a native autoinhibited conformation. Nat Struct Mol Biol 11: 747–755. 15235593 10.1038/nsmb796

[38] Wollman R, Meyer T (2012) Coordinated oscillations in cortical actin and Ca2+ correlate with cycles of vesicle secretion. Nat Cell Biol 14: 261–269. 10.1038/ncb2614 PMC3777337 23143397

[39] Dumitrescu Pene T, Rosé SD, Lejen T, Marcu MG, Trifaró JM (2005) Expression of various scinderin domains in chromaffin cells indicates that this protein acts as a molecular switch in the control of actin filament dynamics and exocytosis. J Neurochem 924: 780–789. 10.1111/j.1471-4159.2004.02907.x 15686479

[40] Wu H, Parsons JT (1993) Cortactin, an 80/85-kilodalton pp60src substrate, is a filamentous actin-binding protein enriched in the cell cortex. J Cell Biol 120: 1417–1426. 7680654 10.1083/jcb.120.6.1417 PMC2119758

[41] Tehrani S, Tomasevic N, Weed S, Sakowicz R, Cooper JA (2007) Src phosphorylation of cortactin enhances actin assembly. Proc Natl Acad Sci U S A 104: 11933–11938. 17606906 10.1073/pnas.0701077104 PMC1924558

[42] Helgeson LA, Nolen BJ (2013) Mechanism of synergistic activation of Arp2/3 complex by cortactin and N-WASP. Elife 2: e00884. 24015358 10.7554/eLife.00884 PMC3762189

[43] Weaver AM, Heuser JE, Karginov AV, Lee WL, Parsons JT, Cooper JA (2002) Interaction of cortactin and N-WASp with Arp2/3 complex. Curr Biol 12: 1270–1278. 12176354 10.1016/s0960-9822(02)01035-7

[44] Siton O, Ideses Y, Albeck S, Unger T, Bershadsky AD, Gov NS, Bernheim-Groswasser A (2011) Cortactin releases the brakes in actin- based motility by enhancing WASP-VCA detachment from Arp2/3 branches. Curr Biol 21: 2092–7. 22169534 10.1016/j.cub.2011.11.010

[45] González-Jamett AM, Momboisse F, Haro-Acuña V, Bevilacqua JA, Caviedes P, et al (2013) Dynamin-2 Function and Dysfunction Along the Secretory Pathway. Front Endocrinol (Lausanne) 4: 126. 24065954 10.3389/fendo.2013.00126 PMC3776141

[46] Sever S, Chang J, Gu C (2013) Dynamin rings: not just for fission. Traffic 14: 1194–1199. 23980695 10.1111/tra.12116 PMC3830594

[47] Cao H, Chen J, Krueger EW, McNiven MA (2010) Src-mediated phosphorylation of dynamin and cortactin regulates the “constitutive” endocytosis of transferrin. Mol Cell Biol 30: 781–792. 19995918 10.1128/MCB.00330-09 PMC2812239

[48] Yamada H, Abe T, Satoh A, Okazaki N, Tago S, et al (2013) Stabilization of actin bundles by a dynamin 1/cortactin ring complex is necessary for growth cone filopodia. J Neurosci 33: 4514–4426. 23467367 10.1523/JNEUROSCI.2762-12.2013 PMC6704951

[49] Samasilp P, Chan SA, Smith C (2012) Activity-dependent fusion pore expansion regulated by a calcineurin-dependent dynamin-syndapin pathway in mouse adrenal chromaffin cells. J Neurosci 32: 10438–10447. 22836276 10.1523/JNEUROSCI.1299-12.2012 PMC3425392

[50] Zhou Z, Misler S, Chow RH (1996) Rapid fluctuations in transmitter release from single vesicles in bovine adrenal chromaffin cells. Biophys J 70: 1543–1552. 8785312 10.1016/S0006-3495(96)79718-7 PMC1225082

[51] Alés E, Tabares L, Poyato JM, Valero V, Lindau M, et al (1999) High calcium concentrations shift the mode of exocytosis to the kiss-and-run mechanism. Nat Cell Biol 1: 40–44. 10559862 10.1038/9012

[52] Guerriero CJ, Weisz OA (2007) N-WASP inhibitor wiskostatin nonselectively perturbs membrane transport by decreasing cellular ATP levels. Am J Physiol Cell Physiol 292: C1562–C1566. 17092993 10.1152/ajpcell.00426.2006

[53] Elhamdani A, Palfrey HC, Artalejo CR (2001) Quantal size is dependent on stimulation frequency and calcium entry in calf chromaffin cells. Neuron 31: 819–830. 11567619 10.1016/s0896-6273(01)00418-4

[54] Egea G, Lázaro-Diéguez F, Vilella M (2006) Actin dynamics at the Golgi complex in mammalian cells. Curr Opin Cell Biol 18: 168–178. 16488588 10.1016/j.ceb.2006.02.007

