# Supplementary figures and images for: Src Kinases Regulate De Novo Actin Polymerization during Exocytosis in Neuroendocrine Chromaffin Cells
Source: PLoS One. 2014 Jun 5;9(6):e99001. doi: 10.1371/journal.pone.0099001 (PMC4047038; doi:10.1371/journal.pone.0099001)

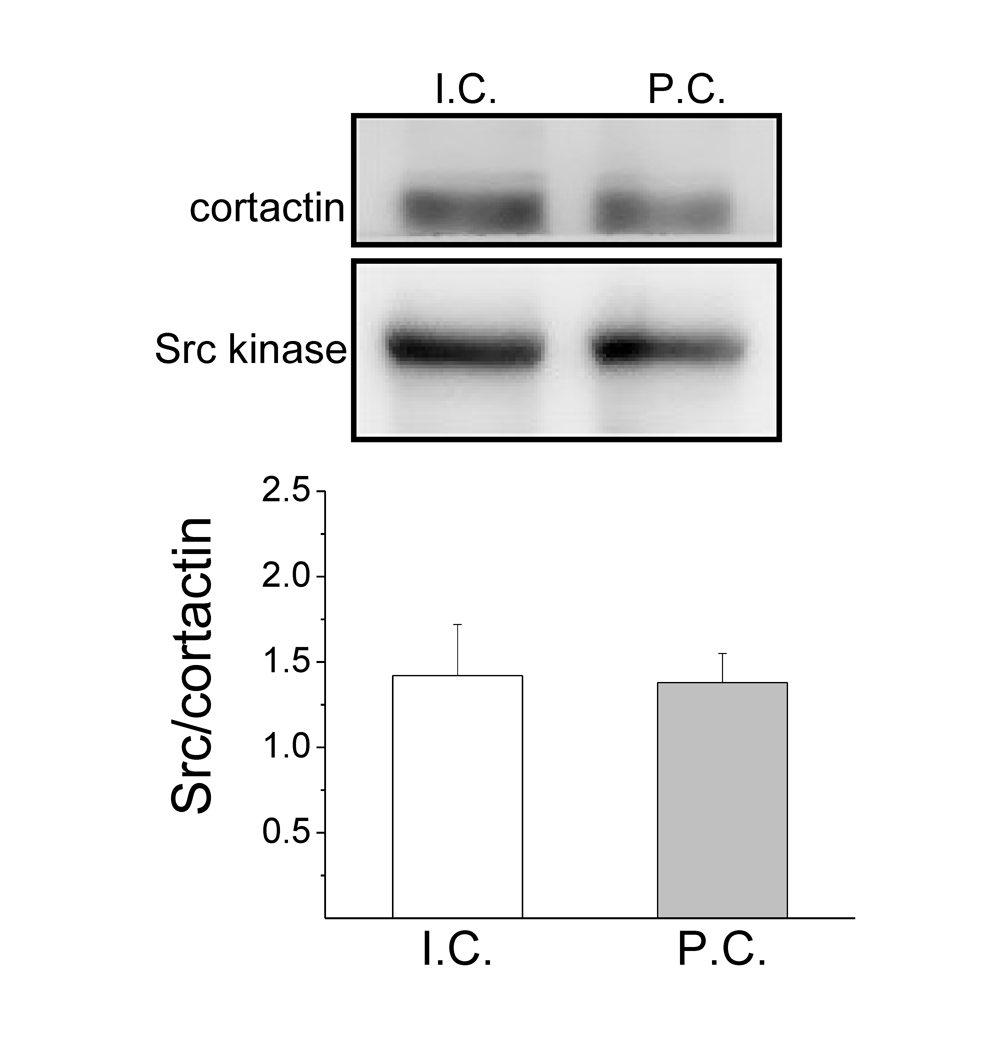

Supplement: Figure S1 — Cell permeabilization does not affect Src kinases/cortactin ratio. In order to determine if Src kinases are lost during permeabilization with digitonin (20 µM for 6 min), we compared the protein levels of Src kinases (60 kDa) with the levels of a protein with a higher molecular weight, cortactin (82 kDa) in total lysates obtained from intact cells (I.C.) and digitonin-permeabilized cells (P.C.). The upper panel shows a representative western blot. The lower panel shows the densitometric analysis of 4 different experiments. The bars represent the average of Src kinases/cortactin ratio. We did not observed differences between the two treatments. (TIF) [file pone.0099001.s001.tif]

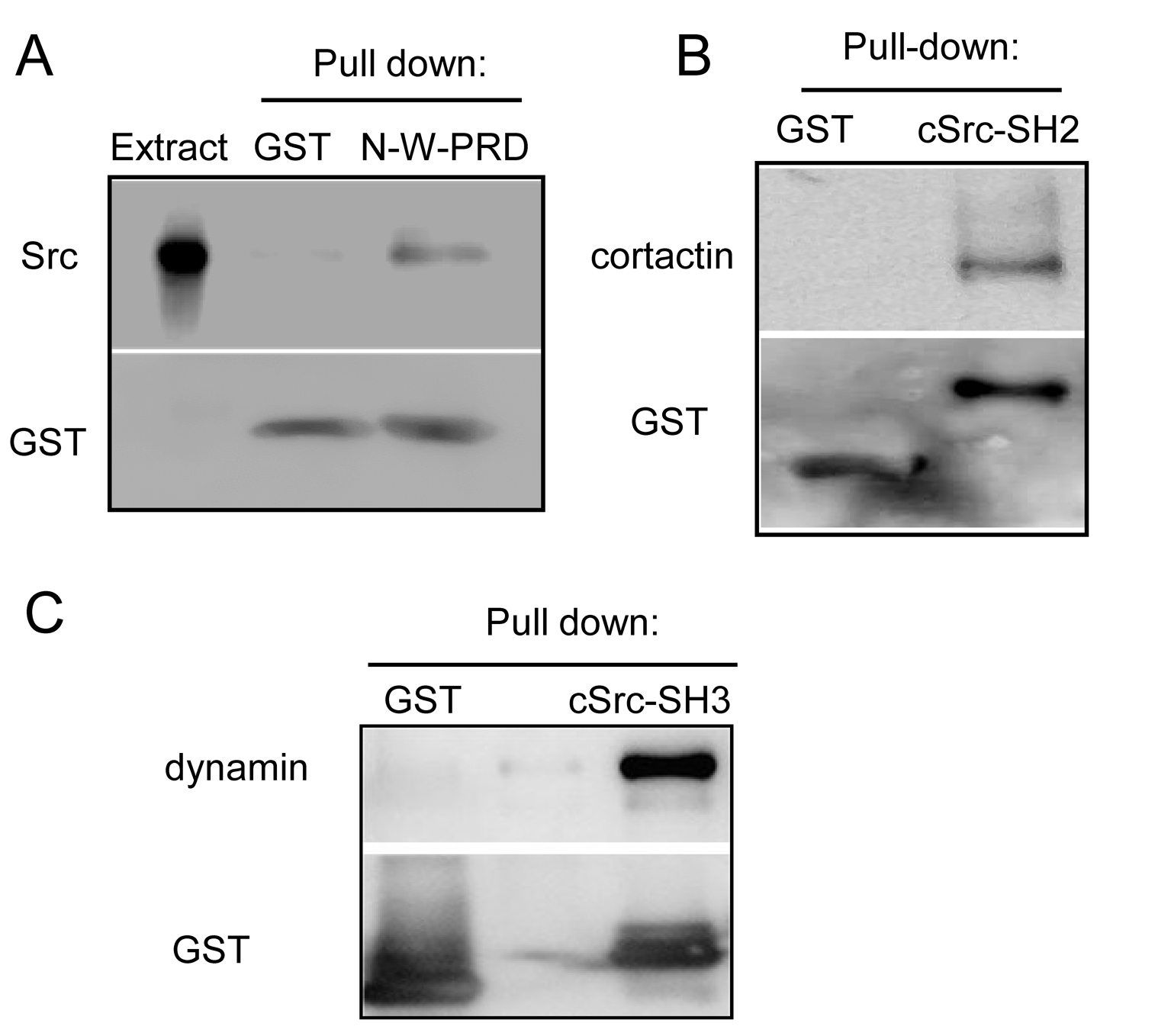

Supplement: Figure S2 — c-Src partners in adrenal chromaffin cells. (A–C) ACC extracts were subjected to pull-down assays using GST, N-WASP PRD-GST (N-W-PRD) (A), c-Src SH2-GST (B) or cSrc SH3-GST (C). Bound proteins were evaluated by immunoblotting using specific antibodies against Src-kinases (A), cortactin (B) dynamin (C) or GST. Note that N-W-PRD pulled-down Src kinases from ACC (A). c-Src-SH2 (B) and c-Src-SH3 (C) efficiently pulled-down cortactin and dynamin from ACC extracts, respectively. (TIF) [file pone.0099001.s002.tif]
