# Supplementary material for: Src Kinases Regulate De Novo Actin Polymerization during Exocytosis in Neuroendocrine Chromaffin Cells
Source: PLoS One. 2014 Jun 5;9(6):e99001. doi: 10.1371/journal.pone.0099001 (PMC4047038; doi:10.1371/journal.pone.0099001)
Supplement: Table S1 — Homology of the amino acid sequences of chicken c-Src SH2 or SH3 domains with the same domains of bovine c-Src, Fyn and c-Yes. For amino acid sequence comparison see: chicken c-Src: Genbank V00402.1, bovine c-Src: UniProt E1BIM8_BOVIN, bovine Fyn: NCBI NM_001077972.1, bovine c-Yes NCBI NM_001101060.1. (DOC) [file pone.0099001.s003.doc]

**Table S1**: Homology of the amino acid sequences of chicken c-Src SH2 or SH3 domains with the same domains of bovine c-Src, Fyn and c-Yes. For amino acid sequence comparison see: chicken c-Src: Genbank V00402.1, bovine c-Src: UniProt E1BIM8­_BOVIN, bovine Fyn: NCBI NM_001077972.1, bovine c-Yes NCBI NM_001101060.1

|  | Chicken c-Src SH2 | Chicken c-Src SH3 |
| --- | --- | --- |
| Bovine c-Src SH2 | 100% |  |
| Bovine c-Src SH3 |  | 100% |
| Bovine Fyn SH2 | 71% |  |
| Bovine Fyn SH3 |  | 79% |
| Bovine c-Yes SH2 | 74% |  |
| Bovine c-Yes SH3 |  | 79% |
