# Supplementary material for: Src Kinases Regulate De Novo Actin Polymerization during Exocytosis in Neuroendocrine Chromaffin Cells
Source: PLoS One. 2014 Jun 5;9(6):e99001. doi: 10.1371/journal.pone.0099001 (PMC4047038; doi:10.1371/journal.pone.0099001)
Supplement: Table S2 — Amperometric parameters of exocytotic events induced by 20 µM ionomycin in cells treated with the Src kinase inhibitor PP2 or its inactive analogue PP3, or injected with c-Src SH2-GST, c-Src SH3-GST or GST alone. Exocytosis was induced with 20 µM ionomycin and monitored by amperometry. Cells were incubated with 20 µM PP2 or its inactive analog PP3 20 min before the exocytosis induction. These agents were kept during the recording. GST, c-Src SH2-GST or c-Src SH3-GST were injected 30 min before cell stimulation. Control corresponds to non-treated cells. Data are means ± SEM of averages. &p<0.05 compared with control; *p<0.05 compared with cells treated with PP3; †p<0.05 compared with cells injected with GST. (DOC) [file pone.0099001.s004.doc]

**Table S2**: **Amperometric parameters of exocytotic events induced by 20 μM ionomycin in cells treated with the Src kinase inhibitor PP2 or its inactive analogue PP3, or injected with c-Src SH2-GST, c-Src SH3-GST or GST alone.** Exocytosis was induced with 20 μM ionomycin and monitored by amperometry. Cells were incubated with 10 μM PP2 or its inactive analog PP3 20 min before the exocytosis induction. These agents were kept during the recording. GST, c-Src SH2-GST or c-Src SH3-GST were injected 30 min before cell stimulation. Control corresponds to non-treated cells. Data are means ± SEM of averages. ^&^p<0.05 compared with control; *p<0.05 compared with cells treated with PP3; ^†^p<0.05 compared with cells injected with GST.

|  | Control | PP3 | PP2 | GST | SH2-GST | SH3-GST |
| --- | --- | --- | --- | --- | --- | --- |
| Number of events | 65.0±7.1 | 76.2±9.9 | 47.2±5.7* | 51.7±7.8 | 40.3±10.1 | 35.5±7.2^&^ |
| Imax (pA) | 98.7±5.0 | 79.1±5.6^&^ | 43.6±2.6*^&^ | 79.4±3.7^&^ | 44.4 ± 4.2^†&^ | 44.2 ± 4.2^†&^ |
| Q (pC) | 1.7±0.1 | 1.8±0.1 | 1.1±0.1*^&^ | 1.5±0.1 | 1.2±0.2^&^ | 1.2±0.1^†&^ |
| t_1/2_ (ms) | 12.9±0.9 | 13.1±0.8 | 19.1±0.9*^&^ | 10.5±0.9 | 30.5±1.0^†&^ | 23.8±1.7^†&^ |
| tP (ms) | 5.2±0.3 | 5.7±0.4 | 9.6±0.6*^&^ | 4.4±0.3 | 11.7±0.6^†&^ | 10.8±0.7^†&^ |
| Foot frequency (%) | 35.6±2.9 | 40.8±4.1 | 54.3±3.1*^&^ | 18.4±3.5^&^ | 51.9±4.1^†&^ | 55.8±4.2^†&^ |
| Foot amplitude (pA) | 8.6±6.8 | 9.9±1.0 | 9.2±0.9 | 6.7±1.2 | 6.4± 0.8 | 7.9±0.7 |
| Foot duration (ms) | 11.2±0.5 | 15.6±1.7 | 22.4±1.9*^&^ | 12.5±2.4 | 17.2±1.3^&^ | 19.9±2.0^†&^ |
| Number of cells | 35 | 15 | 20 | 13 | 12 | 15 |
