# Supplementary material for: Src Kinases Regulate De Novo Actin Polymerization during Exocytosis in Neuroendocrine Chromaffin Cells
Source: PLoS One. 2014 Jun 5;9(6):e99001. doi: 10.1371/journal.pone.0099001 (PMC4047038; doi:10.1371/journal.pone.0099001)
Supplement: Table S3 — Amperometric parameters of exocytotic events cells injected with c-Src SH3-GST and treated with cytochalasin D. Exocytosis was induced with 20 µM ionomycin and monitored by amperometry. Cells injected with 5 µM GST or c-Src SH3-GST (SH3) were incubated with 4 µM cytochalasin D (CytoD) or its vehicle DMSO during 10 min at 37°C before the exocytosis induction and kept during the recording. Data are means ± SEM of averages. *p<0.05 compared with cells injected with GST and treated with DMSO. (DOC) [file pone.0099001.s005.doc]

**Table S3**: **Amperometric parameters of exocytotic events cells injected with c-Src SH3-GST and treated with cytochalasin D.** Exocytosis was induced with 20 μM ionomycin and monitored by amperometry. Cells injected with 5 μM GST or c-Src SH3-GST (SH3) were incubated with 4 μM cytochalasin D (CytoD) or its vehicle DMSO during 10 min at 37 ºC before the exocytosis induction and kept during the recording. Data are means ± SEM of averages. *p<0.05 compared with cells injected with GST and treated with DMSO.

|  | GST+DMSO | SH3+DMSO | GST+CytoD | SH3+CytoD |
| --- | --- | --- | --- | --- |
| Number of events | 47.7±6.1 | 49.5±10.8 | 78.4±10.7* | 38.4±8.7 |
| Imax (pA) | 81.2±8.2 | 59.7±6.0* | 112.6±7.0* | 51.0±4.5* |
| Q (pC) | 1.1±0.1 | 0.9±0.1 | 1.5±0.1* | 1.1±0.1 |
| t_1/2_ (ms) | 11.7±0.7 | 15.3±1.2* | 13.1±0.5 | 20.2±2.4* |
| tP (ms) | 5.3±0.3 | 7.3±0.5* | 6.4±0.3* | 8.6±0.8* |
| Foot frequency (%) | 31.6±2.6 | 40.4±2.9* | 46.2±2.3* | 41.7±3.7* |
| Foot amplitude (pA) | 13.0±1.1 | 12.5±1.2 | 15.4±0.9 | 9.6±0.9* |
| Foot duration (ms) | 14.3±1.0 | 21.3±1.7* | 18.7±0.8* | 21.7±2.7* |
| Number of cells | 15 | 24 | 16 | 14 |
