# Supplementary material for: Src Kinases Regulate De Novo Actin Polymerization during Exocytosis in Neuroendocrine Chromaffin Cells
Source: PLoS One. 2014 Jun 5;9(6):e99001. doi: 10.1371/journal.pone.0099001 (PMC4047038; doi:10.1371/journal.pone.0099001)
Supplement: Table S4 — Amperometric parameters of exocytotic events induced by 20 µM ionomycin in cells treated with wiskostatin. Exocytosis was induced with 20 µM ionomycin and monitored by amperometry. Cells were incubated with 5 µM of the N-WASP inhibitor wiskostatin (Wsk) or the vehicle DMSO. Data are means ± SEM of averages. *p<0.05 compared with cells treated with DMSO. (DOC) [file pone.0099001.s006.doc]

**Table S4**: **Amperometric parameters of exocytotic events induced by 20 μM ionomycin in cells treated with wiskostatin.** Exocytosis was induced with 20 μM ionomycin and monitored by amperometry. Cells were incubated with 5 μM of the N-WASP inhibitor wiskostatin (Wsk) or the vehicle DMSO. Data are means ± SEM of averages. *p<0.05 compared with cells treated with DMSO.

|  | DMSO | Wsk |
| --- | --- | --- |
| Number of events | 43.1±4.3 | 20.4±4.1* |
| Imax (pA) | 100.6± 9.0 | 33.3±4.5* |
| Q (pC) | 1.9±0.2 | 0.6±0.1* |
| t_1/2_ (ms) | 13.0± 0.7 | 13.5±1.7 |
| tP (ms) | 5.9±0.3 | 5.6±0.5 |
| Foot frequency (%) | 48.5±0.4 | 26.8±0.5* |
| Foot amplitude (pA) | 11.7± 0.7 | 7.3±1.2* |
| Foot duration (ms) | 10.5±0.9 | 12.0±2.7 |
| Number of cells | 29 | 15 |
